# Interdependencies and Causalities in Coupled Financial Networks

**DOI:** 10.1371/journal.pone.0150994

**Published:** 2016-03-15

**Authors:** Irena Vodenska, Hideaki Aoyama, Yoshi Fujiwara, Hiroshi Iyetomi, Yuta Arai

**Affiliations:** 1 Metropolitan College, Boston University, 808 Commonwealth Avenue, Boston, MA 02215, United States of America; 2 Center for Polymer Studies, Boston University, 590 Commonwealth Avenue, Boston, MA 02215, United States of America; 3 Graduate School of Sciences, Kyoto University, Kyoto 606-8502, Japan; 4 Graduate School of Simulation Studies, University of Hyogo, Kobe 650-0047, Japan; 5 Department of Mathematics, Niigata University, Niigata 950-2181, Japan; Uppsala University, SWEDEN

## Abstract

We explore the foreign exchange and stock market networks for 48 countries from 1999 to 2012 and propose a model, based on complex Hilbert principal component analysis, for extracting significant lead-lag relationships between these markets. The global set of countries, including large and small countries in Europe, the Americas, Asia, and the Middle East, is contrasted with the limited scopes of targets, e.g., G5, G7 or the emerging Asian countries, adopted by previous works. We construct a coupled synchronization network, perform community analysis, and identify formation of four distinct network communities that are relatively stable over time. In addition to investigating the entire period, we divide the time period into into “mild crisis,” (1999–2002), “calm,” (2003–2006) and “severe crisis” (2007–2012) sub-periods and find that the severe crisis period behavior dominates the dynamics in the foreign exchange-equity synchronization network. We observe that in general the foreign exchange market has predictive power for the global stock market performances. In addition, the United States, German and Mexican markets have forecasting power for the performances of other global equity markets.

## Introduction

Our daily lives are strongly affected by various complex systems such as communication, financial transactions, transportation, just to name a few. The development of modern societies relies on the proper functioning and reliability of these underlying infrastructures. The real world does not function as a set of independent systems but rather of many interdependent systems that interact with each other. Needless to say, our world is becoming more interconnected and any progress or development in one part of the world can be seamlessly exported to another.

Complexity science has been utilized for analyses of interconnectedness in various systems concerning our global world, not the least of which is the international financial and economic complex system. Interdependent network studies [1, 2] have found that coupled networks are more vulnerable to shocks in the system than single isolated networks, and that damage propagates more rapidly in coupled networks than in isolated networks. Thus adverse effects in a coupled system are more severe than in an isolated system.

On the other hand, there are also benefits of connectedness. Among other examples, the effect of membership in intergovernmental organizations, such as the World Trade Organization, quite significantly affects trade relationships among countries. Stronger social and cultural links among countries contribute to stronger influence on economic behavior [3]. When discussing trade relations, one of the important factor for maintaining desirable trade levels is currency stability and keeping the fluctuation of exchange rates within a certain band to avoid disruption in global exports and imports. For our study, social and cultural links in addition to trade relationships might be important for currency management that could be reflected in our quantitative analysis. Another relevant aspect of connectedness is based on the study of cross-border banking flow [4] where the connectedness in 2007 was found not to be extraordinary but rather comparable to earlier peaks. This finding suggests that other factors, rather than the connectedness of the cross-border capital flow, contributed to the severity of the 2008 crisis. Hence, the level of network interdependences is important, and while some connectivity might be beneficial for increasing the stability of the system, too much connectivity can become detrimental [5]. In social context, network interdependencies has been shown to promote cooperation which is out of reach in isolated networks [6]. Economic integration, when one country or currency becomes more dependent on another, and the diversification, when a country or currency is closely related to larger number of countries or currencies, show very different cascading effects through the coupled network [7]. Interconnectedness can increase the vulnerability of the system, when e.g. a financial market experiences significant downfall that cascades to other financial markets. However, the interconnectedness can also offer other channels for funding and can be benefficial when liquidity dries out in certain part of the financial system. Research has shown that a financial network can be most resilient for intermediate levels of risk diversification [8]. So, indeed, depending on the point of view and the nature of the network, connectedness can be both dangerous and beneficial.

A large body of literature studying stock markets and currency markets from various perspectives has emerged as financial markets have changed and cross-border capital flow has increased. The majority of the capital flow increase has been in form of equity investments, with a much smaller portion allocated to overseas loans and fixed income investments. For example, Gagnon and Karolyi [9] report that the total gross capital flows (gross capital purchases and sales between U.S. and foreign investors of U.S. and foreign assets) have increased from less than $100 billion in 1977, corresponding to 1% of U.S. Gross Domestic Product, to over $3.5 trillion, or approximately 30% of U.S. GDP. This significant increase in foreign equity purchases and sales indicates the importance of understanding the relationship between stock markets and foreign exchange markets. Previous studies have investigated the comovements and dependency structures between stock markets and foreign exchange rates [9–21].

Bae, Karolyi and Stulz [15] study international market returns and foreign exchange changes and propose an approach to evaluate contagion in financial markets based on coincidental return shocks in different countries. They use multinominal logistic regression and find that contagion is predictable and depends on interest rates, exchange rate changes, and conditional stock return volatilities. Gagnon and Karolyi [9] suggest that the co-movements in international financial markets are a by-product of increased international cross-border capital flow. According to this study, International markets seem to depend more on other international markets than on country’s domestic fundamentals.

Other studies have focused on stock return predictability in the United States [22–25] and globally (e.g. [26–41]) reporting mixed results, finding positive or negative correlations, stable or non-stable causality relations between markets, and regional and temporal diversity.

Rapach, Strauss and Zhou [40] found that lagged U.S. market returns have been powerful predictor of returns for many other industrialized countries, forecasting returns better than these countries’ economic indicators shedding new light on international predictability. This study uses predictive regression models, pairwise Granger causality tests, and an empirical news diffusion model to analyze how return shocks in one country affect returns in another country. Finally, they also examine the out-of-sample predictive power of lagged U.S. returns and find that out-of-sample gains for numerous non-U.S. countries tend to concentrate during business cycle recessions. These gains have been particularly strong during the most recent Global Financial Crisis (GFC).

The GFC, considered by many to be the worst crisis since the great depression of the 1930s, threatened major financial institutions with the possibility of systemic collapse, propagated value deterioration to financial markets around the world, and involved national governments in bailing out systemically important financial institutions. The crisis also adversely affected housing markets and real estate prices globally and contributed to increased unemployment rates and prolonged workforce unemployment. Ultimately, the global financial meltdown contributed to the European sovereign debt crisis with consequences still lingering in the global financial system. The increased interconnectedness of the financial system up to a certain level could bring stability to the system; however, above a critical point, the system could rapidly become extremely vulnerable, prone to a first-order-like abrupt phase transition [42–44].

Using a network science approach to study the dynamics of financial and economic systems reveals important relationships and defining characteristics within real-world influences that may be overlooked by approaches that do not take into consideration the relationships among the connected parts of the complex financial system. Previous studies related to financial systems [8, 43, 45–49] have shed light not only on the topology of financial networks, i.e., the monetary and information flow within economic and governance networks, but have also examined systemic risk propagation. Acemoglu et al. [50] argue that macroeconomic shocks that originate in one sector may not necessarily be contained close to the origin, but rather could spill over to other parts of the economy affecting other sectors’ outputs generating significant aggregate effect. This study illuminates the often ignored cascading failure effect that could contribute to disastrous consequences in the entire economic system.

The objective of this paper is to study the complex interdependencies and their increasingly interrelated nature of the coupled *global* economic and financial networks, by the use of the latest concepts and methodology from statistical physics and complexity science, to reveal the intrinsic relations in interacting networks. Understanding the interconnectedness and lead-lag relations in the economic complex system could be useful for forecasting the effect of one part of the system on another and potentially offering efficient strategies for system recovery [51].

Unlike previous studies, which have focused on relatively small groups of countries—e.g., G-5, G-7, or the emerging Asian countries, we examine a truly global set of 48 countries, including large and small countries in Europe, the Americas, Asia, and the Middle East. Although we find aspects of regional domination in some local economic communities, using a non-trivial global analysis we also find that these local “economic regions” display network characteristics that are shaped by forces that extend far beyond the local “geographic regions” explored in previous research. The value of our study lies in its use of a novel methodology based on a Complex Hilbert Principal Component Analysis (CHPCA) (see [52–57]) applied to a broad range of economic networks that encompasses more than one world region and more than one characteristic time period.

More specifically, we present a new approach to study the causal relationships between stock and foreign exchange markets. The cross-market relationships that we investigate have significant implications for international risk management and global portfolio management. Understanding the dynamics of the relationship between currency and stock markets is also essential for policy makers. For instance if most of the spillover effects between stock and currency markets are caused by stock market shocks that transfer into currency markets, the policy implications of this phenomenon suggest that a focus on stock-market-centered stability policies might help prevent currency crises. If however shocks propagate from currency to stock markets stabilizing policies for currencies might prove useful in halting or localizing stock market crashes.

In building our analysis we use the CHPCA approach, utilizing the Hilbert transformation and the Rotational Random Shuffling (RRS) methods to identify true comovements, free from contamination of noises, to study the lead-lag relationships between foreign exchange and equity market returns. Our paper offers three methodological advantages over the previous studies: (i) CHPCA enables us to detect beyond-pairwise lead-lag relations as compared with the traditional Granger causality and cross-correlation analysis; (ii) CHPCA is able to extract dynamical correlations simultaneously, while this is difficult to accomplish with PCA; and (iii) RRS provides us with a sound null hypothesis to identify statistically meaningful correlations symultaneously, reaching beyond paiwise or binary relations.

## Materials and Methods

**Fig 1:**
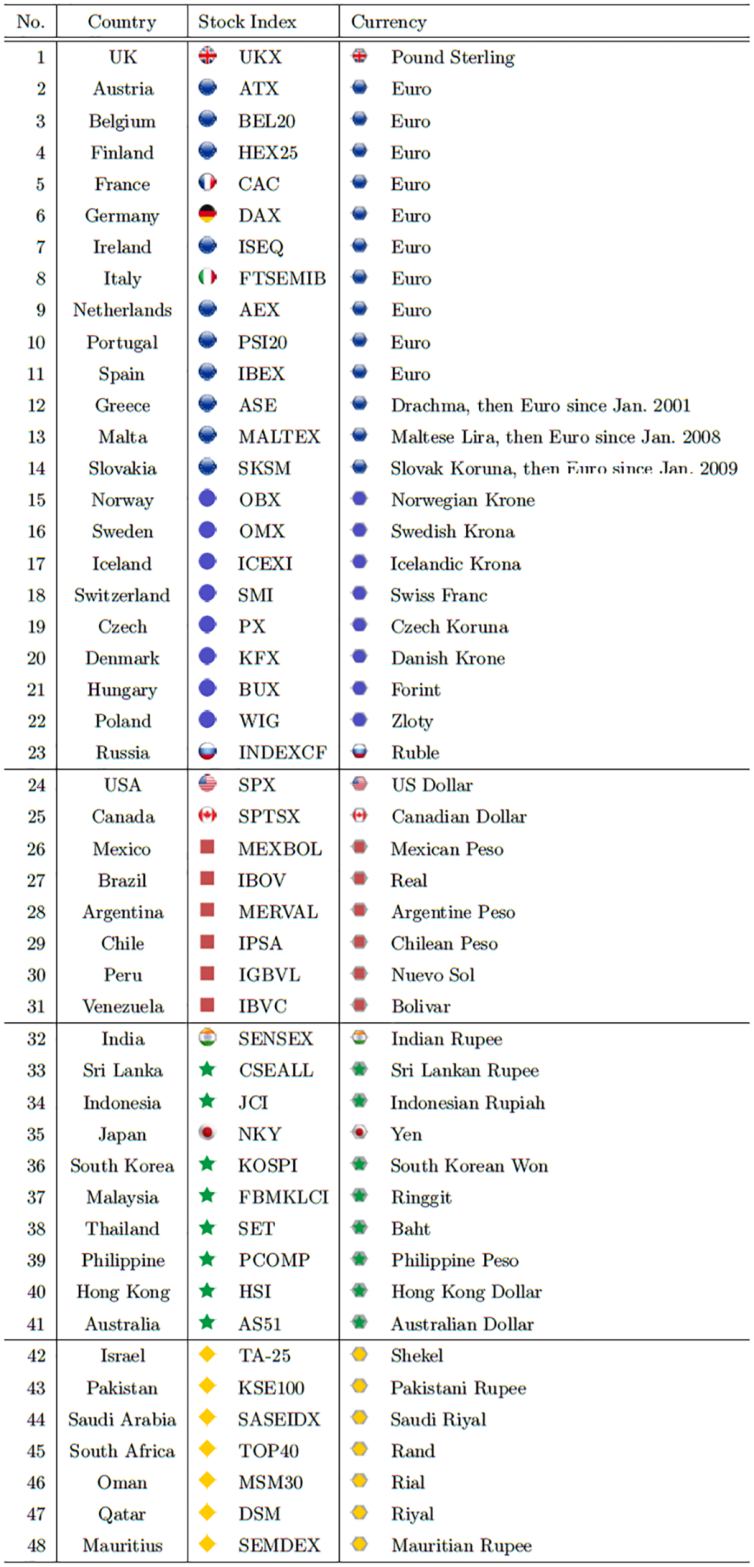
List of 48 countries with their stock-market indices and currencies (with gray hexagonal background). These markers (symbols) are used in the subsequent figures throughout the paper. For the top ten countries (2012 GDP, less China), the markers are given individually with flag motifs, while for others the markers reflect their regionality.

We study daily pricing data for foreign exchange quotes and major stock market indices for 48 countries (see Fig 1) for the 14-year period from Jan. 1, 1999 to Dec. 31, 2012 (5,114 calendar days with 3,652 trading days). We obtain the data from Boston University’s Bloomberg database, which provides financial data for use in academic research.

The time-designation “day” will differ in different parts of the world because of differences in world time zones. Weekends are also missing from the data. However, our methodology is suitable for overcoming these issues, as we discuss later in this section.

We denote this data as *S*_*α*_(*t*), where the node index *α* = 1,2,⋯,48 are the stock-market indices of the 48 countries, *α* = 49,⋯,96 (= *N*) the currencies of the 48 countries in the same order as in Fig 1, and *t* = 1,2,⋯,3652 the date-numbers. The time series we analyze are the log-returns (the logarithm of the growth-rate) *r*_*α*_(*t*) defined by

$$\begin{array}{c} r_{\alpha }\left(t\right):=\log_{10}\;\left[\frac{S_{\alpha }\left(t+1\right)}{S_{\alpha }\left(t\right)}\right]\times \left\{\begin{array}{cc} +1 & \text{for}\;1\leq \alpha \leq 48\\ -1 & \text{for}\;49\leq \alpha \leq 96 \end{array}\right. \end{array}$$ (1)

where *t* runs from 1 to 3651 (≡*T*).

The currency quotes that we analyze are expressed as currency/SDR. (Special drawing right (SDR) is defined as a basket of four major currencies (Japanese Yen, US Dollar, Pound Sterling, and the Euro). The composition of the SDR basket is reviewed every five years by the Executive Board of the International Monetary Fund (IMF) to ensure that it reflects relative importance of currencies in the global financial and trading systems. The last SDR revision took place in November 2010, and the changes became effective on January 1, 2011. According to the latest SDR review in 2015, IMF has decided to incorporate the Chinese Renminbi as the fifth currency in SDR, effective on October 1, 2016.) So when the price thus expressed increases, it means that the currency has depreciated and more “currency” is needed for one SDR. For example, if the USD/SDR price increases, this means that the US Dollar has depreciated.

To avoid confusion and to allow a comparison between the increases (or decreases) in stock market values and the appreciation (or depreciation) of currencies, we transform our original data series from currency/SDR into SDR/currency by multiplying the currency log-returns by (−1).

See Text A in S1 File for summary statistics of the time-series.

We analyze the co-movements of these time series for both equal-time and possible time-delays by applying the Complex Hilbert Principal Component Analysis (CHPCA) (see [52–56]). This is a generalization of the conventional PCA adapted for multivariate time series complexified by the Hilbert transformation.

The CHPCA consists of the following steps:

1. We construct a complex time series by adding the Hilbert transform of the time series as the imaginary component.
2. We do a correlation analysis to obtain the eigenmodes for the period of interest.
3. We do a rotational random shuffling (RRS) simulation to identify which eigenmodes are significant, i.e., which represent co-movements between time series.

We describe each step in turn, features of the method, and then how we can perform community analysis based on the CHPCA.

### A. Hilbert transformation and the complexified time series

We first carry out a discrete Fourier expansion of a time series *r*(*t*) (*t* = 1,2,⋯*T*),

$$\begin{array}{c} r\left(t\right)=\frac{1}{\sqrt{T}}\sum_{k=1}^{T}r^{\left(F\right)}\left(k\right)\;e^{-i\frac{2\pi }{T}kt},\;r^{\left(F\right)}\left(k\right)=\frac{1}{\sqrt{T}}\sum_{t=1}^{T}r\left(t\right)\;e^{i\frac{2\pi }{T}kt}, \end{array}$$ (2)

from which it follows that $r^{\left(F\right)}\left(k\right)=r^{\left(F\right)}{\left(T-k\right)}^{*}$, and that $r^{\left(F\right)}\left(T\right)=\sum_{t=1}^{T}r\left(t\right)/\sqrt{T}$ is real.

For even *T* the Fourier expansion is written as

$$\begin{array}{c} r\left(t\right)=\frac{1}{T}\sum_{t^{\prime }=1}^{T}\left(1+{\left(-1\right)}^{t+t^{\prime }}\right)r\left(t^{\prime }\right)+\frac{2}{\sqrt{T}}\;\text{Re}\left[\sum_{k=1}^{T/2-1}r^{\left(F\right)}\left(k\right)\;e^{-i\frac{2\pi }{T}kt}\right], \end{array}$$ (3)

where the first term in the left-hand side comes from the *k* = *T* and *k* = *T*/2 terms. The Hilbert transform is defined so that it provides the imaginary component of the oscillating part, i.e., the second term on the right-hand side of Eq (3),

$$\begin{array}{c} r^{\left(H\right)}\left(t\right):=\frac{2}{\sqrt{T}}\;\text{Im}\left[\sum_{k=1}^{T/2-1}r^{\left(F\right)}\left(k\right)\;e^{-i\frac{2\pi }{T}kt}\right]. \end{array}$$ (4)

By adding this to the original time series as the imaginary part we obtain a complex time series $\tilde{r}\left(t\right)$,

$$\begin{array}{c} \tilde{r}\left(t\right):=r\left(t\right)+ir^{\left(H\right)}\left(t\right)=\frac{1}{T}\sum_{t^{\prime }=1}^{T}\left(1+{\left(-1\right)}^{t+t^{\prime }}\right)r\left(t^{\prime }\right)+\frac{2}{\sqrt{T}}\;\sum_{k=1}^{T/2-1}r^{\left(F\right)}\left(k\right)\;e^{-i\frac{2\pi }{T}kt}. \end{array}$$ (5)

Similarly we obtain the following equations for odd *T*:

$$r(t)=\frac{1}{T}\sum_{t^{\prime }=1}^{T}r\left(t^{\prime }\right)+\frac{2}{\sqrt{T}}\;\text{Re}\left[\sum_{k=1}^{(T-1)/2}r^{\left(F\right)}\left(k\right)\;e^{-i\frac{2\pi }{T}kt}\right],$$ (6)

$$\tilde{r}\left(t\right):=\frac{1}{T}\sum_{t^{\prime }=1}^{T}r\left(t^{\prime }\right)+\frac{2}{\sqrt{T}}\sum_{k=1}^{(T-1)/2}r^{\left(F\right)}\left(k\right)\;e^{-i\frac{2\pi }{T}kt}.$$ (7)

Note that both Eqs (5) and (7) rotate clockwise in the complex plane.

### B. Complex Correlation Matrix

The normalized log-return ${\tilde{w}}_{\alpha }$ for the complex time series ${\tilde{r}}_{\alpha }\left(t\right)$ is defined by

$$\begin{array}{c} {\tilde{w}}_{\alpha }\left(t\right):=\frac{{\tilde{r}}_{\alpha }\left(t\right)-{\langle {\tilde{r}}_{\alpha }\rangle }_{t}}{\sigma_{\alpha }}, \end{array}$$ (8)

where 〈⋅〉_*t*_ is the average over time *t* = 1,…,*T* (${\langle \cdot \rangle }_{t}:=\left(1/T\right)\sum_{t=1}^{T}\cdot$), and *σ*_*α*_(≥ 0) is the standard deviation of ${\tilde{r}}_{\alpha }$ over time,

$$\begin{array}{c} \sigma_{\alpha }^{2}:=\frac{1}{T}\sum_{t=1}^{T}|\;{\tilde{r}}_{\alpha }\left(t\right)-{\langle {\tilde{r}}_{\alpha }\rangle }_{t}{|}^{2}=\langle |{\tilde{r}}_{\alpha }{|}^{2}\rangle_{t}-{\left|{\langle {\tilde{r}}_{\alpha }\rangle }_{t}\right|}^{2}. \end{array}$$ (9)

The complex correlation matrix $\tilde{C}$ is an *N* × *N* (*N* = 96) Hermitian matrix defined as

$$\begin{array}{c} {\tilde{C}}_{\alpha \beta }:={\langle {\tilde{w}}_{\alpha }{\tilde{w}}_{\beta }^{*}\rangle }_{t}, \end{array}$$ (10)

whose diagonal elements are 1 by definition of the normalized log-return ${\tilde{w}}_{\alpha }$ (Eq (8)).

The eigenvalues λ^(*n*)^, which are non-negative due to the chirality of the Hermitian matrix $\tilde{C}$, and the corresponding eigenvectors ***V***^(*n*)^ of the matrix $\tilde{C}$ satisfy the following relations,

$$\begin{array}{cc} & \tilde{C}\;V^{\left(n\right)}=\lambda^{\left(n\right)}V^{\left(n\right)}, \end{array}$$ (11)

$$\begin{array}{cc} & V^{(n)*}\cdot V^{\left(m\right)}=\delta_{nm}, \end{array}$$ (12)

$$\begin{array}{cc} & \sum_{n=1}^{N}\lambda^{\left(n\right)}=N, \end{array}$$ (13)

$$\begin{array}{cc} & \tilde{C}=\sum_{n=1}^{N}\lambda^{\left(n\right)}V^{\left(n\right)}V^{(n)†}. \end{array}$$ (14)

The superscripts in parentheses such as (*n*) denote the indices of different eigenvalues and eigenvectors, having the range *n* = 1,…, *N*. We order the eigenvalues in descending order λ^(*n*)^ ≥ λ^(*n*−1)^, for any *n*. Here *δ*_*nm*_ is the Kronecker delta, i.e., *δ*_*nm*_ = 1 if *n* = *m*, *δ*_*nm*_ = 0 if *n* ≠ *m*. Eigenmodes with large eigenvalues are the key to uncovering co-movements in this set of time series. This is the case because when the time series are expanded in terms of the eigenvectors,

$$\begin{array}{c} {\tilde{w}}_{\alpha }\left(t\right)=\sum_{n=1}^{N}a^{\left(n\right)}\left(t\right)\;V_{\alpha }^{\left(n\right)}, \end{array}$$ (15)

the mode-signals *a*^(*n*)^(*t*) satisfy

$$\begin{array}{c} {\langle a^{(n)*}a^{\left(m\right)}\rangle }_{t}=\delta_{nm}\lambda^{\left(n\right)}, \end{array}$$ (16)

which can be proven using Eqs (10), (11) and (12). Eq (16) shows that the larger the eigenvalue the larger will be the presence of the eigenvector, with their mean strength proportional to the square root of the eigenvalues. The next question is how to determine which eigenmodes (with large eigenvalues) are significant, i.e., which are free from noise.

Because in our case *T* is odd, we expand the normalized log-return ${\tilde{w}}_{\alpha }$ as

$$\begin{array}{c} {\tilde{w}}_{\alpha }\left(t\right)=\frac{2}{\sqrt{T}}\sum_{k=1}^{(T-1)/2}w_{\alpha }^{\left(F\right)}\left(k\right)\;e^{-i\frac{2\pi }{T}kt}, \end{array}$$ (17)

in a way similar to that in Eq (7). Substituting this into Eq (10), we find that

$$\begin{array}{c} {\tilde{C}}_{\alpha \beta }:=\frac{4}{T}\sum_{k=1}^{(T-1)/2}w_{\alpha }^{\left(F\right)}\left(k\right)w_{\beta }^{\left(F\right)}{\left(k\right)}^{*}=\frac{4}{T}\sum_{k=1}^{(T-1)/2}\left|w_{\alpha }^{\left(F\right)}\left(k\right)w_{\beta }^{\left(F\right)}{\left(k\right)}^{*}\right|e^{i(\delta_{\alpha }\left(k\right)-\delta_{\beta }\left(k\right))}, \end{array}$$ (18)

where *δ*_*α*_(*k*) is the phase of $w_{\alpha }^{\left(F\right)}\left(k\right)$. Thus when there is only one Fourier-component in either ${\tilde{w}}_{\alpha }\left(t\right)$ or ${\tilde{w}}_{\beta }\left(t\right)$, the phase of the complex correlation coefficient ${\tilde{C}}_{\alpha \beta }$ is equal to *T*/(2*πk*) times the amount of *delay* of the time series ${\tilde{w}}_{\alpha }\left(t\right)$ relative to ${\tilde{w}}_{\beta }\left(t\right)$ (in that Fourier mode). If there are multiple Fourier components, the phase of the complex correlation coefficient ${\tilde{C}}_{\alpha \beta }$ is a weighted (non-linear) average of the time-delay as in Eq (18). When *T* is even, a similar relation holds.

### C. Rotational Random Shuffling (RRS) method

As explained in detail in [58], several methods can be used to establish which eigenmodes are important when extracting significant information from related time series. Random matrix theory (RMT) results for the spectrum of iid (independent, identically distributed) time series [59] clearly indicate that a time series that is truly random induces a nontrivial spectrum of eigenvalues. The RMT method is not suitable for our purpose, however, (i) because each of the time series analyzed possesses non-negligible autocorrelation leading to spurious cross-correlations and (ii) because 12 European countries (#2 through #11 in Fig 1) adopted the Euro in January 1999 and their currency time series is thus identical by definition. Because the drachma was closely related to the Euro before Greece (#12 in Fig 1) adopted the Euro in 2001, its currency time series are very close to those of the other European countries. Because of these characteristics in our data, there are eleven zero eigenvalues and one eigenvalue close to zero, and the constraint in Eq (13) may give rise to artificially large eigenvalues. Also we remark the RMT spectrum formula is valid only for random matrices of infinite dimensions. Such mathematical ideality thereby restricts the applicability of RMT as a null hypothesis to detect statistically meaningful eigenmodes in CHPCA dealing with random matrices of finite dimensions.

We thus use the RRS simulation proposed in [57, 60], not RMT, to extract a meaningful spectrum of significant eigenvalues. The RRS method is free from the limitations of RMT as described above. In this simulation we first randomly rotate each time series (with the currencies of 11 European countries from Austria to Greece in Fig 1) as

$$\begin{array}{c} {\tilde{w}}_{\alpha }\left(t\right)\to {\tilde{w}}_{\alpha }\left(\text{Mod}\left(t-\tau_{\alpha },T\right)+1\right), \end{array}$$ (19)

where

$$\begin{array}{c} \tau_{\alpha }=\left\{\begin{array}{cc} 0, & \text{for}\;\alpha =50,\cdots 60,\\ \text{(pseudo-)random}\;\text{integer}\;\in [0,N], & \text{otherwise}. \end{array}\right. \end{array}$$ (20)

We then obtain eigenvalues λ^(*n*)^ from the rotated time series. In this way we keep the autocorrelation of individual time series intact but cancel out co-movements between the time series (except the trivial co-movements among the 11 European countries that use the common Euro currency). Thus by comparing the resulting shuffled eigenvalue spectrum with the original eigenvalue set we can identify the important, non-random co-movements within the equity-currency coupled network.

### Advantages of the CHPCA

This methodology is suitable for efficiently extracting significant lead-lag relationships among currencies and financial markets. The CHPCA efficiently identifies significant lead-lag relationships in the global financial network that occur due to the difference of a “day” in different parts of the world as well as lead-lag relationships due to real time-delay in the reactions of one country to economic phenomena in other countries. Computational efficiency is especially important since we analyze a large dataset consisting of 96 time-series. One may apply ordinary cross-correlation analysis by shifting each time-series and maximizing the correlation coefficients as functions of time-shifts. However, doing so for all the possible pairs with only several possibilities for the trial time-shift values will be quite a daunting task. For example, allowing a six-day delay between a pair will require us to examine 6*N*(*N* − 1)/2 ≃ 2.7 × 10^4^ cases. Granger causality methodology reduces the complexity of calculations by including more than one lag at one time in its regression analysis, but is still characterized as a pairwise analysis. Besides the issue of computational efficiency, furthermore, those methodologies are not appropriate for our targeted network in which nodes are strongly coupled to each other. For instance, one is not able to fully understand the three-body problem by shedding light only on binary relations. To elucidate the multiple relationships in a simultaneous manner, one may think of applying the conventional PCA to time-series each shifted, e.g. with 6 possible time shifts as our pairwise example above. Such an analysis, however, would bring about 6^*N*^ ≃ 5 × 10^74^ cases, an astronomical amount of calculations! In contrast, the CHPCA offers tremendous efficiencies, identifying significant lead-lag relationships with just one calculation, as demonstrated in the next section. In addition, the eigenvectors associated with significant eigenvalues, offer insight into different comovement modes in the overall network, not limited to pairwise relationships.

### Community Analysis

A promising way of studying the time-averaged aspects in the collective behaviors of the financial constituents is to derive a network from our CHPCA results and explore the formation of distinct network communities. For this purpose, we first note the spectral decomposition of the correlation coefficients,

$$\begin{array}{c} \tilde{C}=\sum_{n=1}^{N}\lambda^{\left(n\right)}V^{\left(n\right)}V^{(n)†}. \end{array}$$ (21)

We then define the filtered complex correlation coefficients,

$$\begin{array}{c} {\tilde{C}}_{\alpha \beta }^{\left(N_{s}\right)}=\sum_{n=1}^{N_{s}}\lambda^{\left(n\right)}V_{\alpha }^{\left(n\right)}V_{\beta }^{(n)†}=r_{\alpha \beta }\;e^{i\theta_{\alpha \beta }}. \end{array}$$ (22)

Namely, ${\tilde{C}}_{\alpha \beta }^{\left(N_{s}\right)}$ is a correlation coefficient obtained by retaining only the *N*_*s*_ dominant eigenvalues (*N*_*s*_ = 6 for the entire period and period three and *N*_*s*_ = 5 for periods one and two). Variables *r*_*αβ*_ and *θ*_*αβ*_ are the magnitude and phase of the correlation coefficient, respectively.

We consider the correlation matrix ${\tilde{C}}^{\left(N_{s}\right)}$ as an adjacency matrix, and construct a network of the financial nodes linked to each other with the corresponding correlation coefficients as (complex) weights.

The network thus constructed is in principle a complete graph in which all pairs of nodes are connected. However the coupling strength between nodes *α* and *β* varies with their associated magnitude *r*_*αβ*_ ranging from 0 to 1. Note that the linkage has direction depending on the lead-lag relation between the two nodes: *β* (*α*) leads *α* (*β*) if *θ*_*αβ*_ takes a positive (negative) value. Here we define the directed links of each pair to be between the leader and the follower. The in-degree $k_{\alpha }^{\text{in}}$ and the out-degree $k_{\alpha }^{\text{out}}$ of node *α* are hence calculated as

$$\begin{array}{c} k_{\alpha }^{\text{out}}=\sum_{\beta (\theta_{\alpha \beta }<0)}r_{\alpha \beta }\;. \end{array}$$ (23)

$$\begin{array}{c} k_{\alpha }^{\text{in}}=\sum_{\beta (\theta_{\alpha \beta }>0)}r_{\alpha \beta }\;, \end{array}$$ (24)

By calculating

$$\begin{array}{c} \Delta k_{\alpha }=k_{\alpha }^{\text{out}}-k_{\alpha }^{\text{in}}\;, \end{array}$$ (25)

we can identify different lead-lag relations among the financial constituents as shown in the next section.

To shed light on synchronization in those economic relations, we further impose the following condition on each pair of nodes *α* and *β* we want to connect:

$$\begin{array}{c} |\theta_{\alpha \beta }|<\theta_{c}\;, \end{array}$$ (26)

where *θ*_*c*_ is a cutoff angle to be specified. By assuming weight of the link between the nodes is given by the magnitude *r*_*αβ*_ we are able to construct a synchronization network in which nodes moving in phase are linked to each other.

Here we detect communities of comoving nodes in our financial networks thus constructed by maximizing a quality function known as modularity [61]. This is a prevailing method to detect communities in complex networks [62]. The modularity *Q* for an unweighted network with *M* links which is decomposed into *L* communities is defined as

$$\begin{array}{c} Q=\sum_{s=1}^{L}\left[\frac{e_{s}}{M}-{\left(\frac{d_{s}}{2M}\right)}^{2}\right], \end{array}$$ (27)

where *e*_*s*_ and *d*_*s*_ are the total number of internal links and the sum of degree of nodes within community *s*, respectively. The value of *L* is also simultaneously determined through the modularity maximization. The modularity measures the fraction of links within the given communities of a network in reference to the expected fraction of the intralinks if the network is randomized with the degree of each node preserved. For the modularity of weighted networks such as those studied here, one may generalize Eq (27) by replacing the unit weight of each link by its actual weight [63]; for instance, the degree of each node is the total sum of weights of its connecting links. To carry out maximization of the modularity, we employ the fast unfolding method [64]. Since it is a computational method of stochastic nature, we repeat the procedure using different series of random numbers to obtain 10,000 partitions and select the result with the largest modularity.

Visualization is another useful tool to illuminate structural properties of complex networks. To have an optimized layout for each of our networks, we adopt a spring-electrical model in which pairs of nodes with direct links are physically connected with springs and any pairs of nodes repel each other through a repulsive Coulomb force [65]. The attractive force due to the spring keeps tightly connected nodes close in a space. On the other hand, the repulsive Coulomb force tends to distribute nodes uniformly over the available space and to prevent entanglement of the network. The optimization for the network layouts were then obtained by gradually cooling “temperature” of the fictitious system to zero temperature through molecular dynamics simulations [66].

## Results and Discussion

### Interdependent Network Analysis

**Fig 2:**
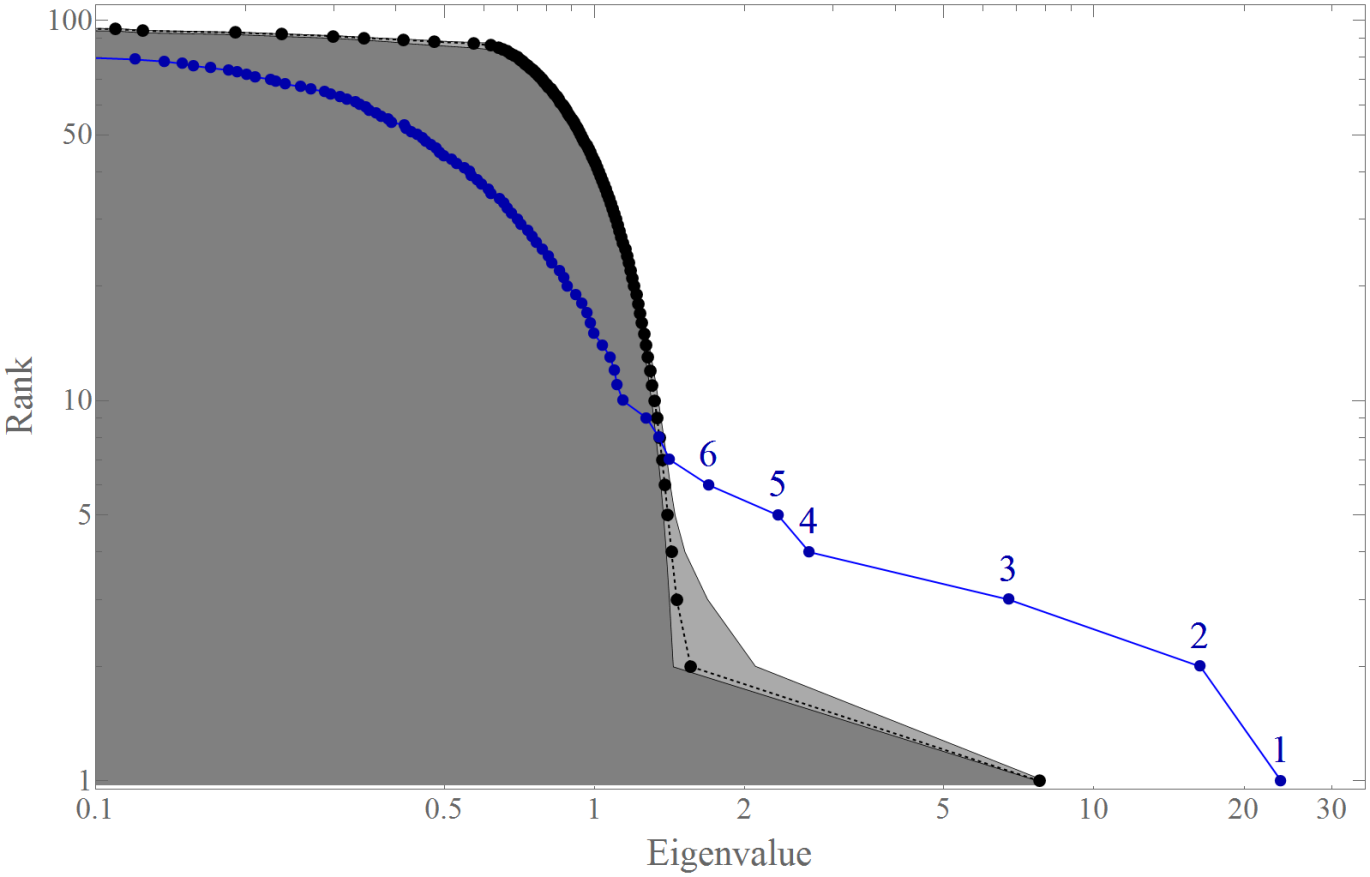
Significant eigenvalues identified by the CHPCA with RRS method. The blue dot denoted “*n*” shows the *n*-th eigenvalue λ^(*n*)^ (*x*-axis) and the eigenvalue rank (*y*-axis). The gray small dots and the lighter gray area show the average RRS and the 99% range. The six largest eigenvalues are clearly outside of their RRS ranges, and show significant relationships in the interdependent network.

Using CHPCA methodology, we obtain eigenvalues λ^(*n*)^ and the corresponding eigenvectors ***V***^(*n*)^ where *n* = 1⋯96 and λ^(1)^ > λ^(2)^ > ⋯ > λ^(96)^. The eigenvalues (the large dots with numbers and the blue line) and the RRS results (the small dots and the dashed line) are plotted in Fig 2 and displayed in Table 1. The first six eigenvalues, λ^(1)^⋯λ^(6)^, lie outside the RRS 99% error-range and thus are clearly identifiable. These six largest eigenvalues can be used to explain the significant co-movements in the system. For periods 1–3, similar analyses show that 5 CHPCA eigenvectors are significant (see Text B and its first figure (Fig B1) in S1 File).

**Table 1:** List of CHPCA Eigenvalues, their 99% RRS Range, and the mean contribution rate $\sqrt{\lambda^{\left(n\right)}/\lambda^{\left(1\right)}}$. Although the seventh eigenmode is outside the RRS range, we exclude this mode as insignificant as it is very close to the boundary.

*n*	λ^(*n*)^	99% RRS range	$\sqrt{\lambda^{\left(n\right)}/\lambda^{\left(1\right)}}$
1	23.74	$7.79_{-0.02}^{+0.17}$	1
2	16.35	$1.56_{-0.12}^{+0.54}$	0.83
3	6.76	$1.46_{-0.05}^{+0.22}$	0.53
4	2.69	$1.42_{-0.03}^{+0.09}$	0.37
5	2.33	$1.40_{-0.03}^{+0.05}$	0.31
6	1.69	$1.38_{-0.02}^{+0.03}$	0.27
7	1.41	$1.36_{-0.02}^{+0.03}$	0.24
8	1.35	$1.35_{-0.02}^{+0.02}$	0.24

**Fig 3:**
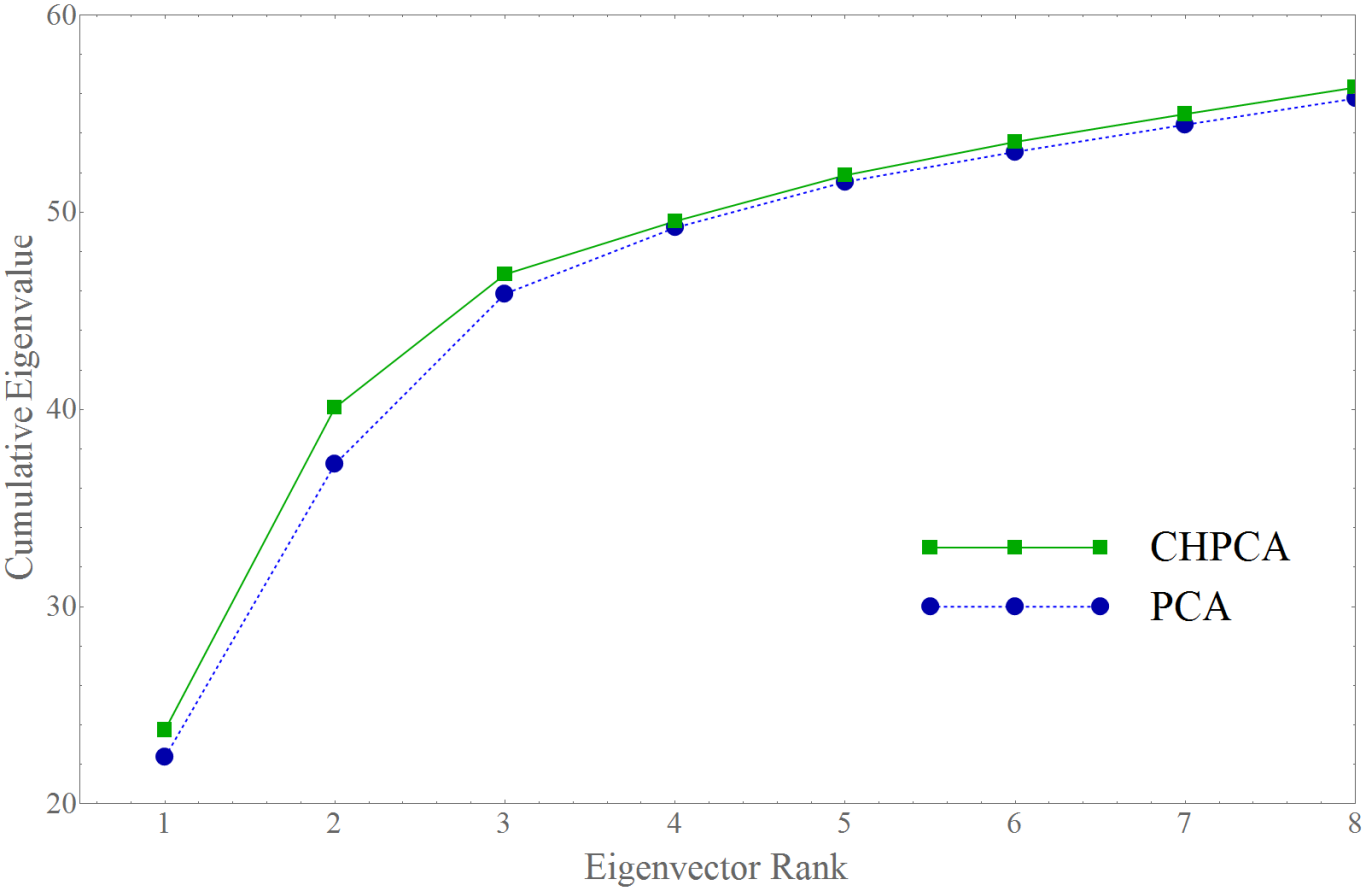
Comparison between the PCA and CHPCA eigenvalues. In each case, the partial sum of the eigenvalues, $\sum_{n=1}^{K}\lambda^{\left(n\right)}$ (*y*-axis) versus *K* (*x*-axis), for PCA (blue and dashed) and for CHPCA (green and solid). The fact that the CHPCA sums of eigenvalues are always above PCA-based eigenvalue sums shows that CHPCA is a stronger analytic tool in identifying important co-movements.

As discussed above, because CHPCA is able to detect cross-correlations with lead-lag relations in multivariate time series it should, in theory, be superior to a conventional PCA-based analysis. Fig 3 shows that, in practice, CHPCA is in fact superior to PCA and that the cumulative sum of the CHPCA eigenvalues is consistently larger than that of the PCA eigenvalues.

#### Properties of the dominant eigenmodes

**Fig 4:**
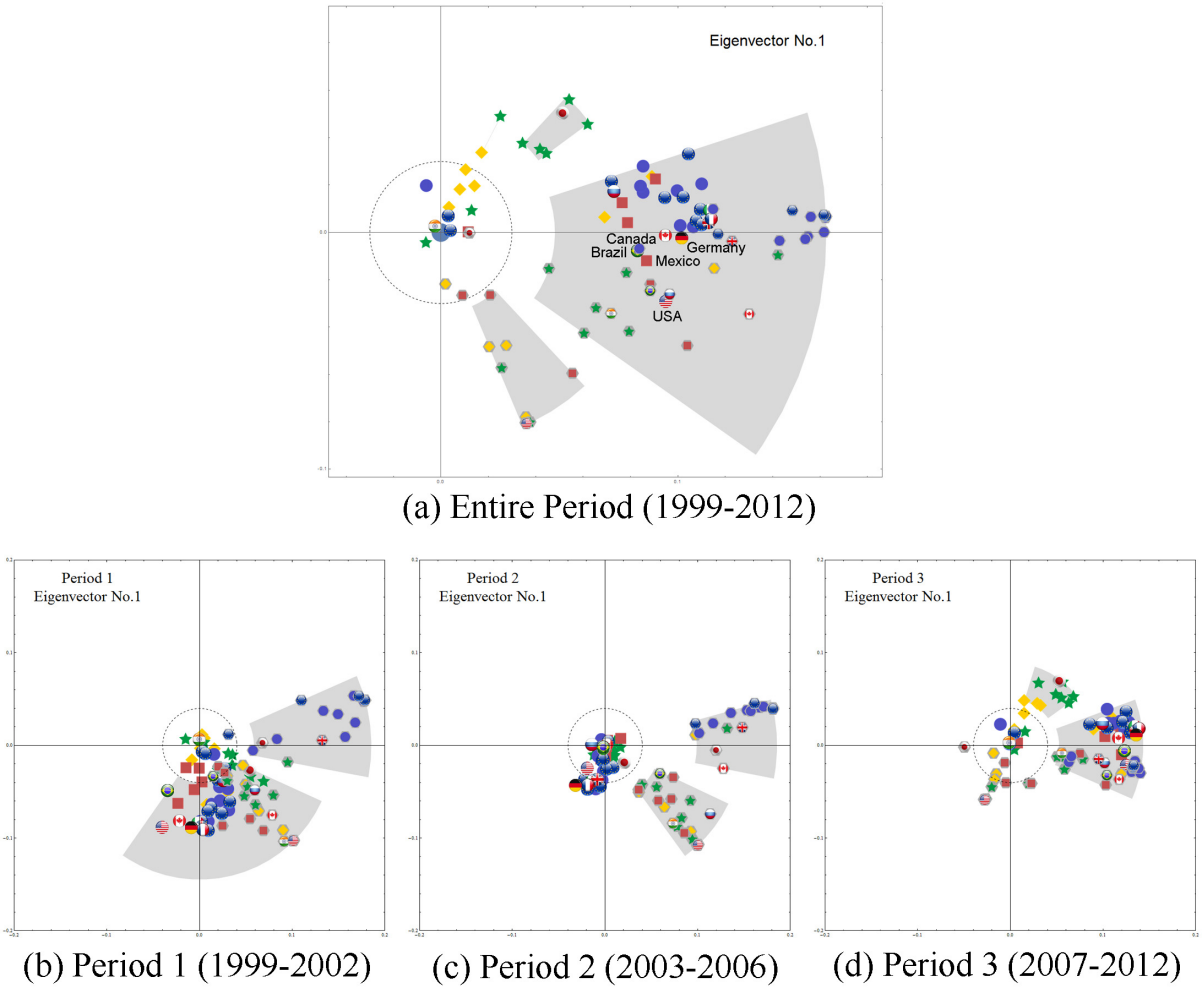
The first eigenvector (whose eigenvalue is the largest) components on a complex plane. The flags without the gray background represent the equity markets, while the dark gray hexagons behind the flags represent the currencies as shown in Fig 1. The nodes with absolute value larger than 0.04 (which is indicated with a dashed circle) are grouped so that nodes with phase gap smaller than 0.2 [rad] are in the same group. Panel (a) for the entire period and (b)–(d) for the three subperiods. See main body of the text for interpretations of the results.

**Fig 5:**
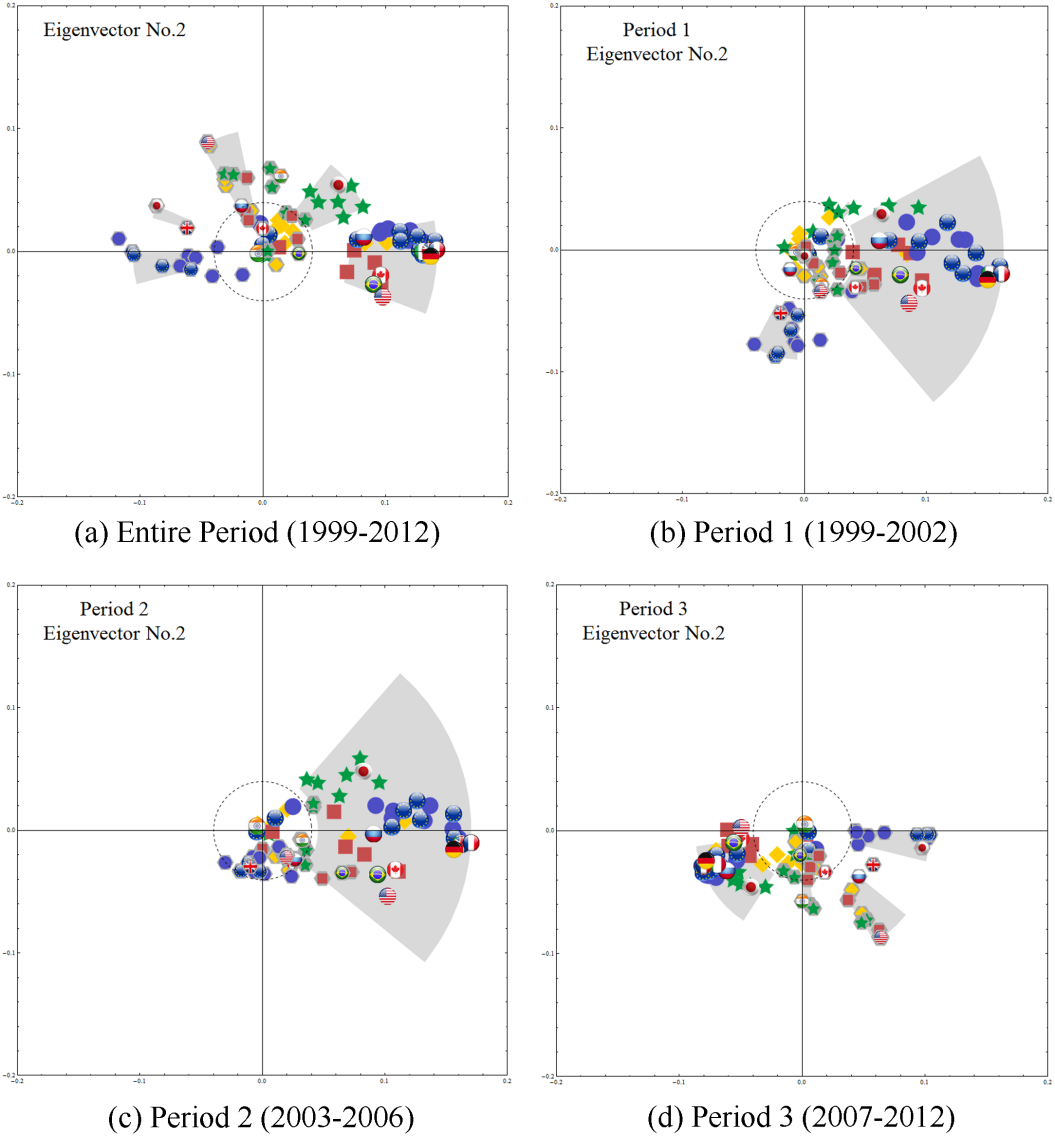
The components of the second eigenvector. We multiply the second eigenvector components by the ratio of strength of the corresponding mode-signals, $\sqrt{\lambda^{\left(2\right)}/\lambda^{\left(1\right)}}$, reflecting the fact that the contribution of the *n*-th eigenmode to the time series is proportional to $\sqrt{\lambda^{\left(n\right)}}$. For the entire period, the currency markets are distributed to the left of the origin, mainly close to the negative real axis, while equity markets are along the positive real axis and also in the first quadrant, indicating negative equal-time correlation between the equity and foreign exchange markets. Again, these features are almost the same for Period 3, the severe crisis period, and differ in other periods.

**Fig 6:**
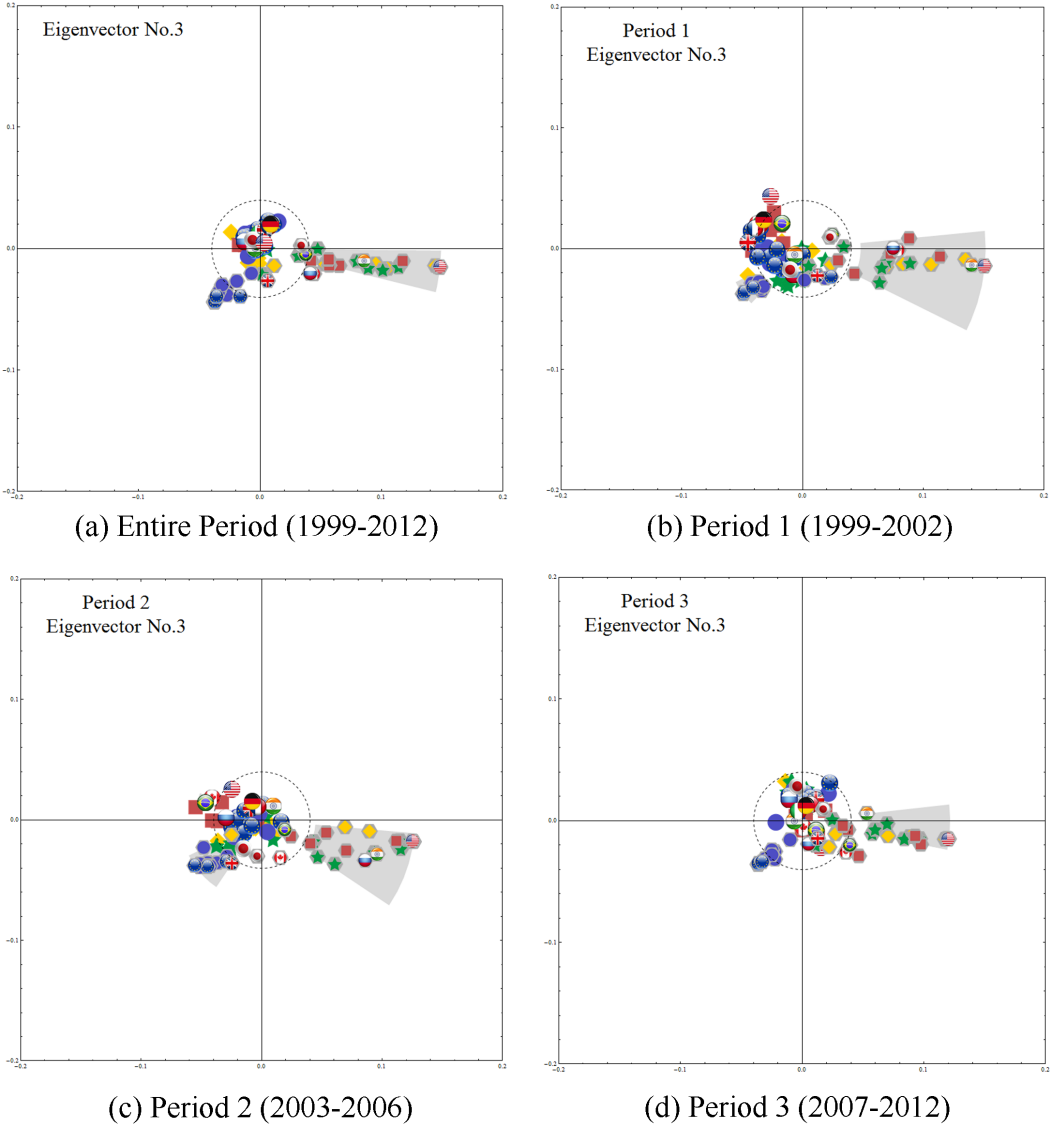
The third eigenvector components. For the entire period and all subperiods, we multiply the third eigenvector components by $\sqrt{\lambda^{\left(3\right)}/\lambda^{\left(1\right)}}$ and observe two large clusters, one containing most of the European currencies and the other the Asian, South American, and Middle Eastern currencies, largely dominated by the US dollar. Note that here the correlations among stock market indices are almost non-existent.

Using CHPCA analysis, we identify the dynamics of the eigenvector components of the six largest eigenvalues, we plot the first three ***V***^(1)^⋯***V***^(3)^ in Figs 4–6, and observe the following:

Fig 4 shows the causal relationship between the stock markets and the currencies in the 48 countries we analyze in this study during the entire 1999–2012 period. The behavior of the eigenvector components corresponding to the largest eigenvalues indicates that currency performance usually leads or influences stock market performance in each of the countries.

The US, Mexican and Brazilian equity markets, closely followed by the German and Canadian equity markets, are predictive of the performances of equity markets in other countries. During the 1999–2002 mild crisis period the US, Germany, and UK equity markets are also leaders in forecasting currency returns.

During the calm 2003–2006 period financial markets do not exhibit strong correlations but currency markets do. Asian and South American currencies as well as the US dollar are predictive of the performance of the Euro, British Pound, Canadian Dollar, and Japanese Yen.

The severe 2007–2012 crisis period strongly resembles the entire 1999–2012 period, indicating that this period strongly influences market trends and currency market-equity market interdependencies throughout the period. Findings based on largest-eigenvalue analysis agree with the importing firm (or country) theory, i.e., that an appreciation in a local currency lowers the price of imported goods to the firm or country and increases profits realized in future payables to the firm or country—the payables being denominated in foreign currency—and thus increases the equity value of the importing firm or country. This in turn has a positive impact on equity markets.

Fig 5 shows the behavior of the eigenvector components corresponding to the second largest eigenvalue. Equity markets and currency markets exhibit an equal-time (concurrent) negative correlations, and this is particularly strong in the US, German, UK, and Swiss markets. Other European countries such as Norway, Sweden, Hungary and Czech Republic show similar behavior between their respective equity markets and currencies. There are also strong geographical positive correlations in which separate clusters are formed by the European and Asian equity markets. Note also that Asian equity markets are predictive of their corresponding currency returns.

During the 1999–2002 mild crisis period currency markets are predictive of equity market performance. During the calm 2003–2006 period this predictive power of currency markets is somewhat diminished. The severe 2007–2012 crisis period exhibits behavior similar to the entire analyzed period, once again dominating the equity-currency causal relationship that we observe for the entire 1999–2012 period.

The results for the second largest eigenvalue show that positive performances in the US, German, UK, Swiss as well as other European markets are frequently linked to a simultaneous depreciation of their respective currencies. Although we would expect a local currency to be in higher demand when an equity market increases and thus appreciate, increases in the equity market can also be driven by domestic (not international) investors. Other factors can also affect equity and currency markets, including quantitative easing, inflation, and fluctuating interest rates. Coupling a weaker currency with a strong equity market is a tactic advocated by the trade-dominant theory in which a government protects its currency by artificially depreciating it in order to boost exports and maintain stability in the foreign exchange market.

The results for Asia and South America indicate that when the performance of a country’s financial markets is positive, the country’s currency appreciates (with a time lag) because the desirable investment climate has increased demand for the currency by investors following a balanced portfolio strategy [17].

Fig 6 shows the relationship among markets explained by the third eigenvector components. For the entire period and all subperiods (Fig 6(a)–6(d)), we observe two large clusters, one containing most of the European currencies and the other the Asian, South American, and Middle Eastern currencies, largely dominated by the US dollar. Note that here the correlations among stock market indices are almost non-existent.

#### Insights from smaller significant eigenmodes

In the previous section we examined the dominant relationships between stock markets and currencies within the first, second, and third eigenvectors. In our network analysis, however, we have identified a total of six significant eigenvalues. The last column of Table 1 shows that the smaller eigenvalues (4–6) contribute much less (approximately 30% of the first eigenmode) to explaining currency-equity network dynamics. Thus *on average* the fourth, fifth, and six eigenmodes are much less important than the first three.

Note the proximity of the fourth and fifth eigenvalues, which means that these eigenmodes are approximately *degenerate* and that any linear combination of these two eigenvectors are close to being one eigenvector. Thus a separate examination of the fourth and fifth eigenvectors may produce no useful results.

**Fig 7:**
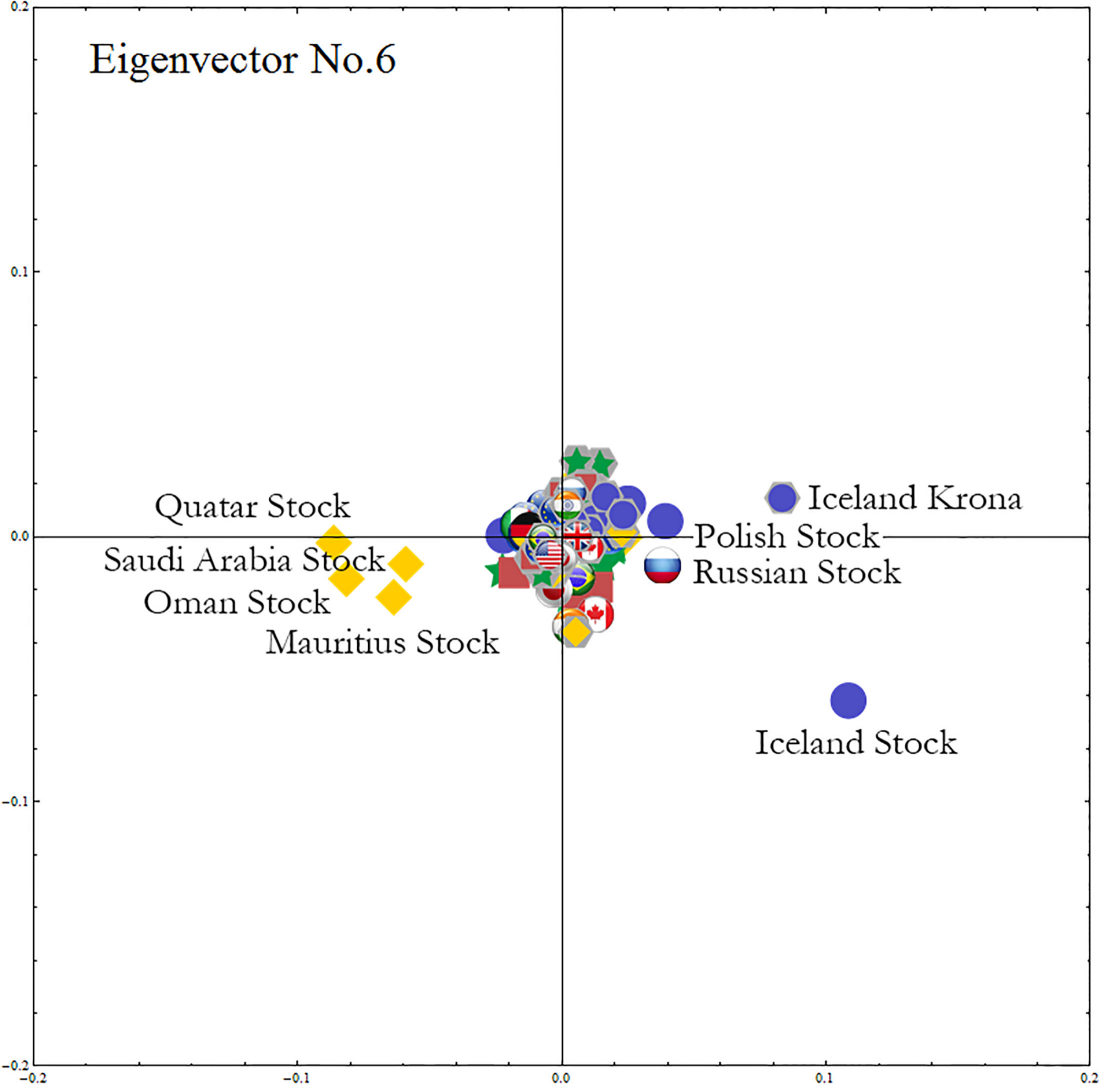
The sixth eigenvector components. We multiply the sixth eigenvector components by $\sqrt{\lambda^{\left(6\right)}/\lambda^{\left(1\right)}}$. We include the names for the nodes that have large absolute values for the entire period. In this eigenmode the largest contribution comes from the Iceland’s stock market, followed by the Icelandic krona. Closely following Iceland, are Polish and Russian stock markets with positive correlation and oil exporting countries’ stock markets with negative correlation.

Fig 7 shows the sixth eigenvector components that dominate the dynamics of the stock and foreign exchange market behavior, including the Icelandic Krona and the Icelandic, Polish, and Russian stock markets on one side and the Middle Eastern stock markets on the other.

**Fig 8:**
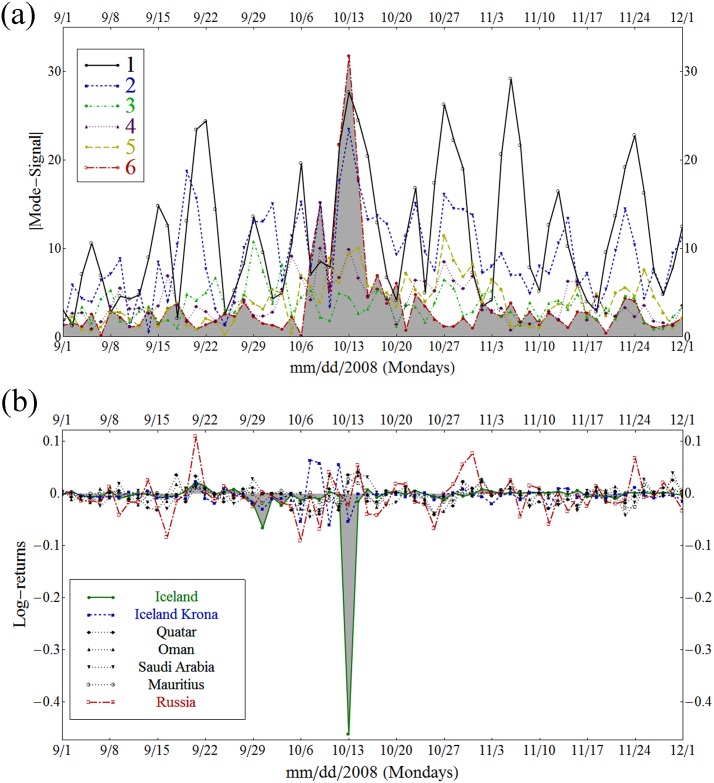
Log-returns and mode-signals of the sixth eigenvector selected components. (a) Absolute values of the mode-signals for the eigenmodes 1 to 6 from September 1st to December 1st of 2008 (The ticks for the x-axis are given only for Mondays). The red dots connected with red dash-dot lines show that the sixth eigenmode, evidently significant on October 13th at the height of the Icelandic banking crisis, exceeds the contribution of the first eigenmode. (b) Behavior of the log-returns of the time series that have large absolute values in the sixth eigenvector. This behavior of the actual time series is consistent with the large mode-signal of the sixth eigenmode observed in panel (a).

Fig 8(a) also shows that on October 13, 2008, at the onset of Icelandic banking crisis, this sixth eigenmode has a stronger influence than even the first eigenmode. As we saw in Fig 7, the sixth eigenmode is singular in the sense that while its average contribution to the whole set of the time series is small (as $\sqrt{\lambda^{\left(6\right)}/\lambda^{\left(1\right)}}≃0.24$ in Table 1), it has a limited number of nodes with large absolute values lead by Iceland’s stock market and the Icelandic Krona. In this plot we observe that this sixth eigenmode has large contribution in the time-signal at the time of Iceland’s financial crisis, even exceeding that of the first eigenmode. Fig 8(b) shows that the log-return of the Icelandic stock market (the green line with the shaded gray area) dips sharply. On this day the Icelandic stock market lost over 90% of its market capitalization and contributed to the collapse of all three of Iceland’s major financial institutions, creating unprecedented social unrest and initiating a major economic and political crisis. According to *The Economist* of December 11, 2008, relative to the size of its economy Iceland’s collapse was the largest in economic history [67].

This demonstrates the strength of our CHPCA and RRS based analysis and its sensitivity to such world events as the Icelandic financial crisis. Although it was localized in time and occurred in only a couple of time series out of 96, our analysis did not exclude this occurrence as noise and was able to identify this Icelandic stock market event as a signal of importance in the sixth eigenmode.

### Results of Community Analysis

In this section we summarize our community analysis findings and offer economic interpretations of the results.

**Fig 9:**
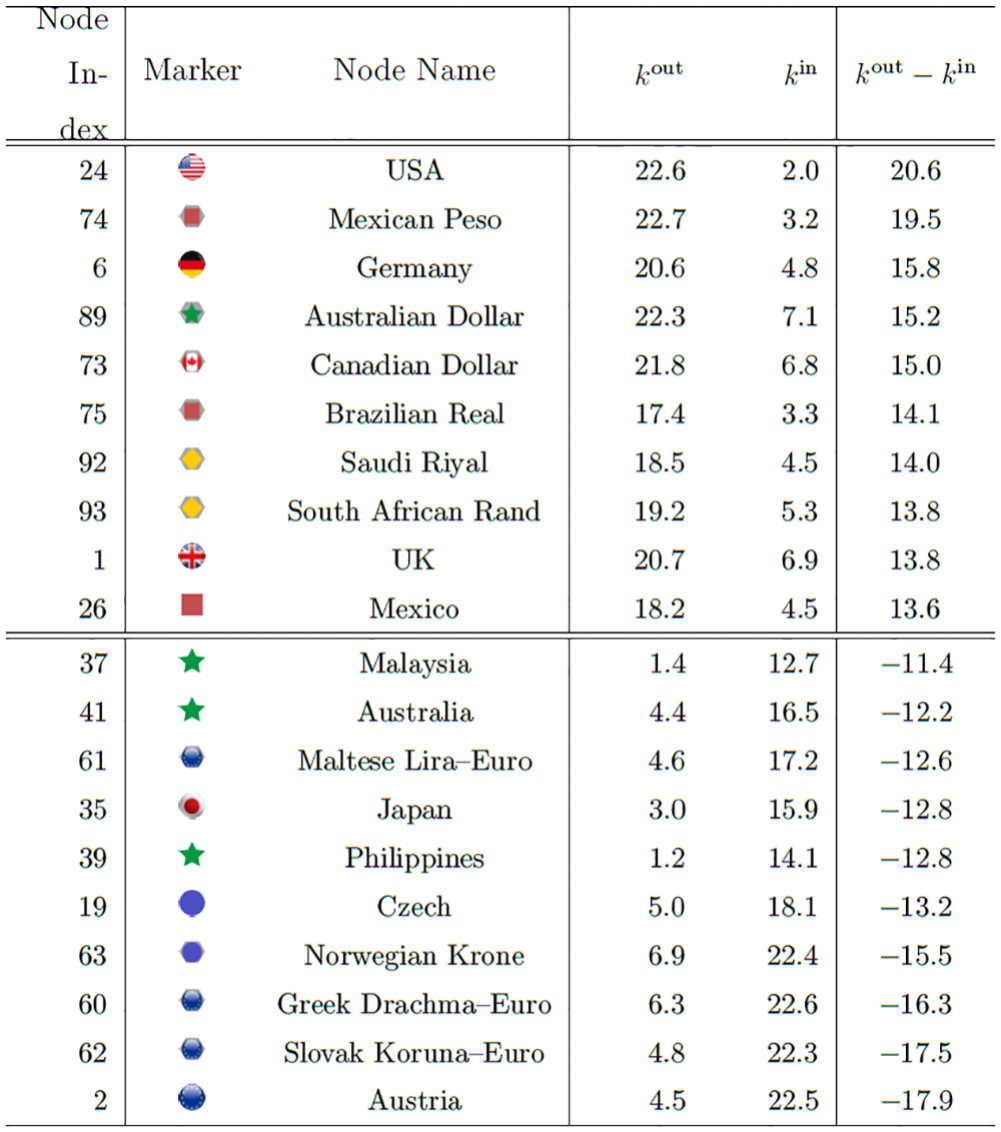
Top 10 and bottom 10 nodes by *k*^out^−*k*^in^ for the entire period (1999–2012). Note that node indices larger than 48 are for currencies; for example, node index 74 is the currency for Mexico, or country #26 (= 74−48) in Fig 1.

**Fig 10:**
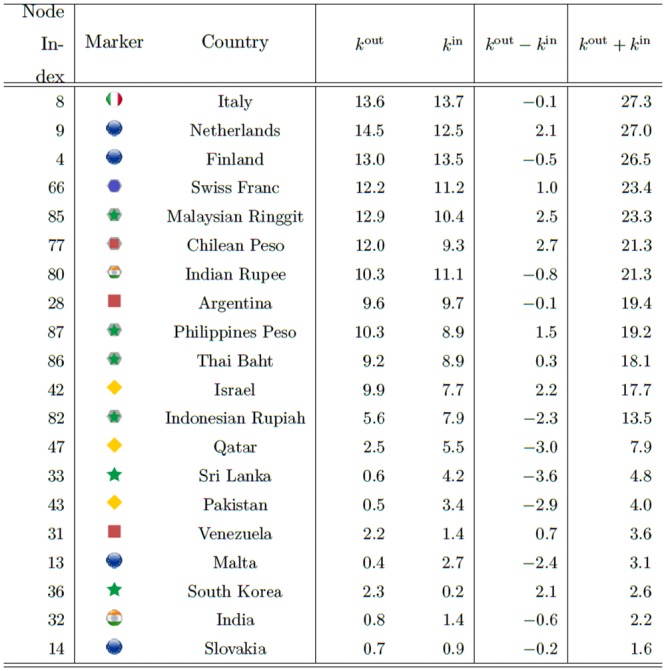
Stock markets and currencies with the lowest absolute differences |*k*^out^−*k*^in^| for the entire period (1999–2012). We have ordered the components in descending order of (*k*^out^ + *k*^in^) to show the relative position of stock markets and currencies in the coupled network.

By calculating the difference between the out-degree and in-degree of each node, namely Eq (25), we can single out four typical cases for nodes with different lead-lag relations, (i) $k_{\alpha }^{\text{out}}\gg k_{\alpha }^{\text{in}}$, (ii) $k_{\alpha }^{\text{out}}⪡k_{\alpha }^{\text{in}}$ (see the first and second half of Fig 9 respectively), (iii) $k_{\alpha }^{\text{out}}≃k_{\alpha }^{\text{in}}≃0$, and (iv) $k_{\alpha }^{\text{out}}≃k_{\alpha }^{\text{in}}≃0$ (see Fig 10). The stock markets and currencies satisfying condition (i) lead the world economy. Those satisfying condition (ii) follow the leaders. The nodes satisfying condition (iii) are sometimes leaders and sometimes followers, and those satisfying condition (iv) are nodes that are for the most part isolated from the rest of the world.

Fig 9 shows that the US and the German stock markets and the Mexican Peso and the Australian Dollar are the strongest leaders in the stock market-foreign exchange coupled network during the 1999–2012 period. Note that the Mexican Peso co-moves closer to the US market than the US dollar because during this entire period the Mexican Peso and the US stock market are peripheral to their communities, while the US dollar is a core node of the community to which the Mexican Peso belongs. In period 3, the Mexican Peso and the US market are identified as members of the same community. During this period the Euro and some of the other European currencies are the ones most influenced by the rest of the global stock markets and currencies.

Fig 10 shows that the extent to which the Italian, Dutch, and Finish stock markets are influenced by other markets and currencies are similar to the extent to which they influence other stock markets and currencies. The Slovakian, Indian, and South Korean markets are the most isolated from the rest of the world, and neither significantly affect other markets nor are influenced by them. For a more elaborate listing of leaders and followers in the coupled forex-stock market network for the entire period (1999–2012) and the three subperiods (1999–2002), (2003–2006), and (2007–2012), see [68].

During the 1999–2002 and 2003–2006 periods the US and German stock markets exhibit the largest Δ*k* values and lead the world economy. During the 2007–2012 period, however, they are replaced by the currencies of such developing countries as Mexico, South Africa, and Brazil. On the other hand, during the 1999–2002 and 2003–2006 periods the European currencies are followers that exhibit large negative Δ*k* values, the most affected during 1999–2002 being the Greek Drachma (prior to the adoption of the Euro in 2001), the Hungarian Forint, and the Czech Republic Koruna, and the most affected during 2003–2006 the Slovak Koruna (prior to the adoption of the Euro in 2009), the Norwegian krone, and the Swedish Krona. During the 2007–2012 period stock markets generally follow currency markets, with the stock markets of Austria, Philippines, and Hungary exhibiting the largest negative Δ*k* values.

#### Community structure

Community detection, the extraction of nodes tightly connected as communities, is widely used to identify clustering structures in complex networks. Here a community is a collection of co-moving nodes. Setting *θ*_*c*_/*π* = 0.1 in Eq (26), we obtain the synchronization networks in the entire period and in the three subperiods. Maximizing the modularity and identifying the network communities, we find that the network has a meaningful community structure in each period with modularity values of 0.415 (1999–2012), 0.516 (1999–2002), 0.556 (2003–2006), and 0.348 (2007–2012). In practice, modularity values exceeding approximately 0.3 indicate that community decomposition is significant [61]. Details of the community detection algorithm are described in Text D in S1 File, including the sensitivity of the community structure to different cutoff values for *θ*_*c*_.

**Fig 11:**
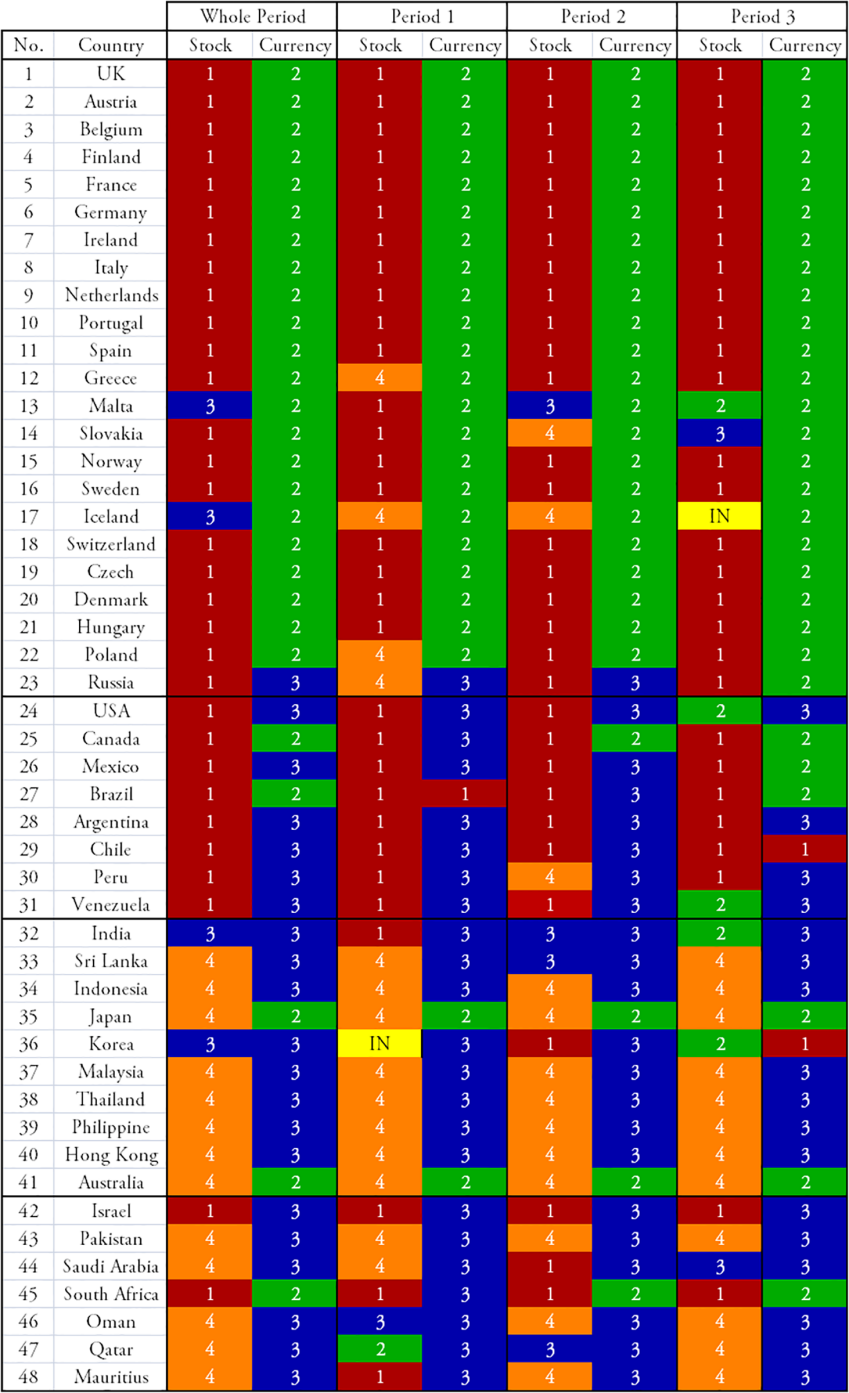
Results of the community detection for the entire period (1999–2012) and for the three characteristic periods (period 1: 1999–2002, period 2: 2003–2006, and period 3: 2007–2012). The stock markets and currencies of 48 countries are decomposed into four co-moving communities, designated by numbers (“IN” means an independent node), in each period.

**Fig 12:**
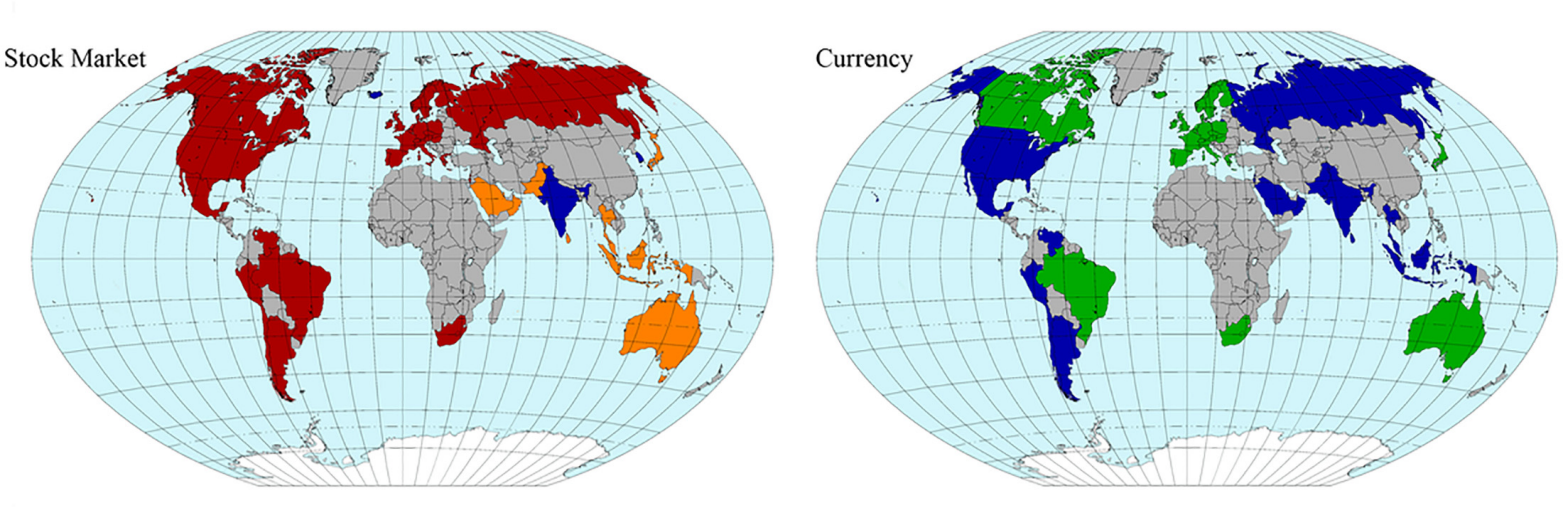
Geographical distribution of the community structure for the entire period given in Fig 11 including the communities dominated by stock markets with red and orange colors and communities dominated by mainly currencies with blue and green colors.

We can clearly see the community structure in Figs 11 and 12. In the following sections we will denote the community #*n* in Fig 11 as C*n*.

Note that the community structure of the stock market and foreign exchange coupled network is relatively stable over both the entire period and the three sub-periods, i.e., the 1999–2002 (mild crisis), 2003–2006 (calm period), and 2007–2012 (severe crisis) periods. We thus classify the financial constituents into four dominant communities:

1. The stock market community C1, dominated by Europe, South America, the USA, and Canada as major categories that always appear in this community;
2. The currencies-only community C2, dominated by Europe and Canada;
3. The currency community C3, dominated by Russia, the USA, South America, Asia, and the Middle East; and
4. The stock market community C4, dominated by Asia (including Japan) and the Middle East.

Figs 11 and 12 show that almost all of the countries of North and South America belong to one stock-market dominated community, and that the Middle East, Asia and Australia belong to another stock-market dominated community. In contrast, there is one currency community that encompasses the US dollar, a number of Latin American currencies, the Middle East, Russia, India, and several smaller Asian countries, and another currency community that encompasses the Canadian dollar, the Brazilian Real, the Australian dollar, the Euro, and the South African Rand.

**Fig 13:**
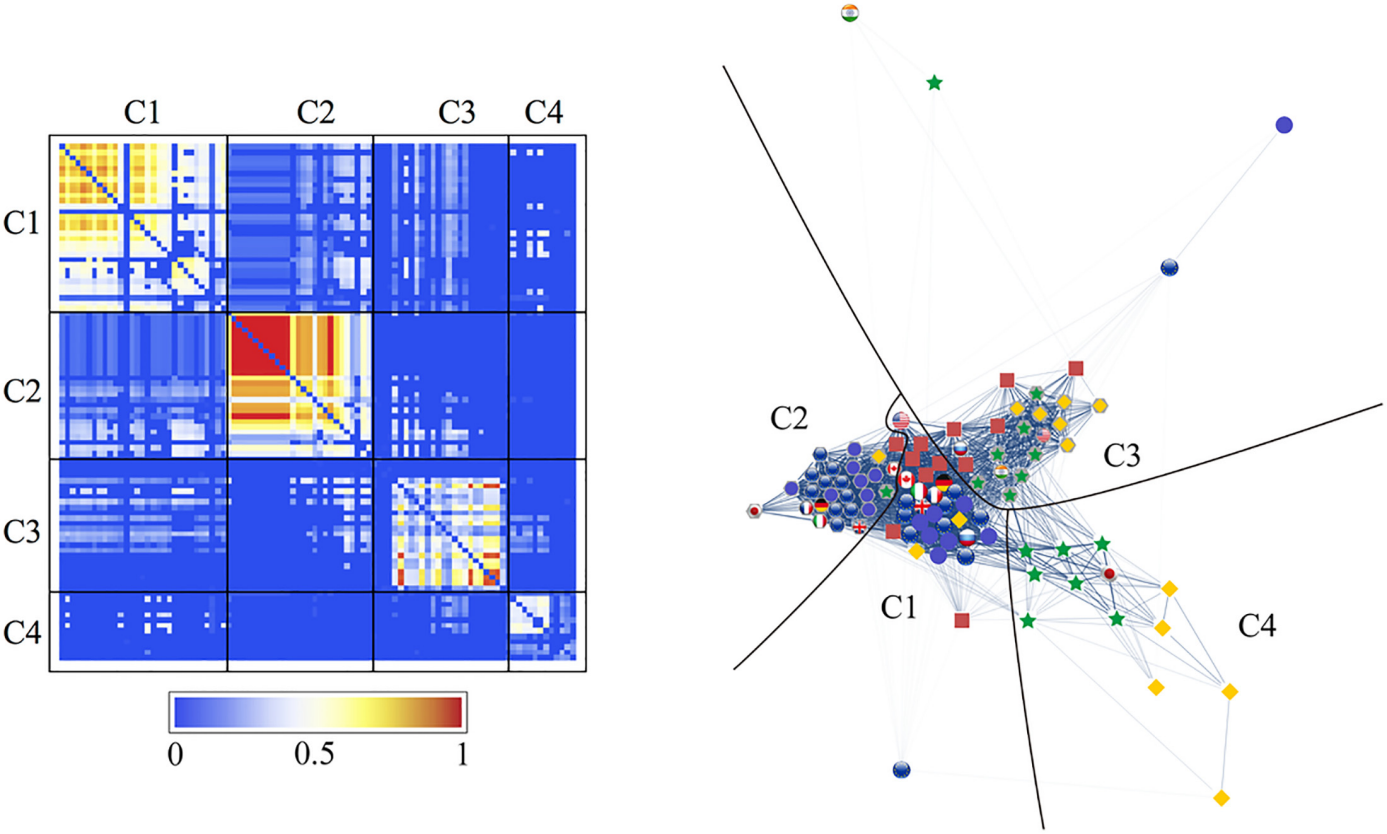
Community structure for the financial network for the entire period (1999–2012). We constructed the network with *θ*_*c*_/*π* = 0.1. The left panel shows the adjacency matrix sorted according to the classification into four communities of synchronizing nodes: the first community (C1) is a group of stock markets mainly in European and American countries; the second community (C2), a euro-based currency group; the third community (C3), a group of currencies represented by the U.S. dollar; the fourth community (C4), an Asian stock market group surrounding Japan. The right panel shows an optimized layout of the network in an spring-electrical model, with boundaries separating the communities.

**Fig 14:**
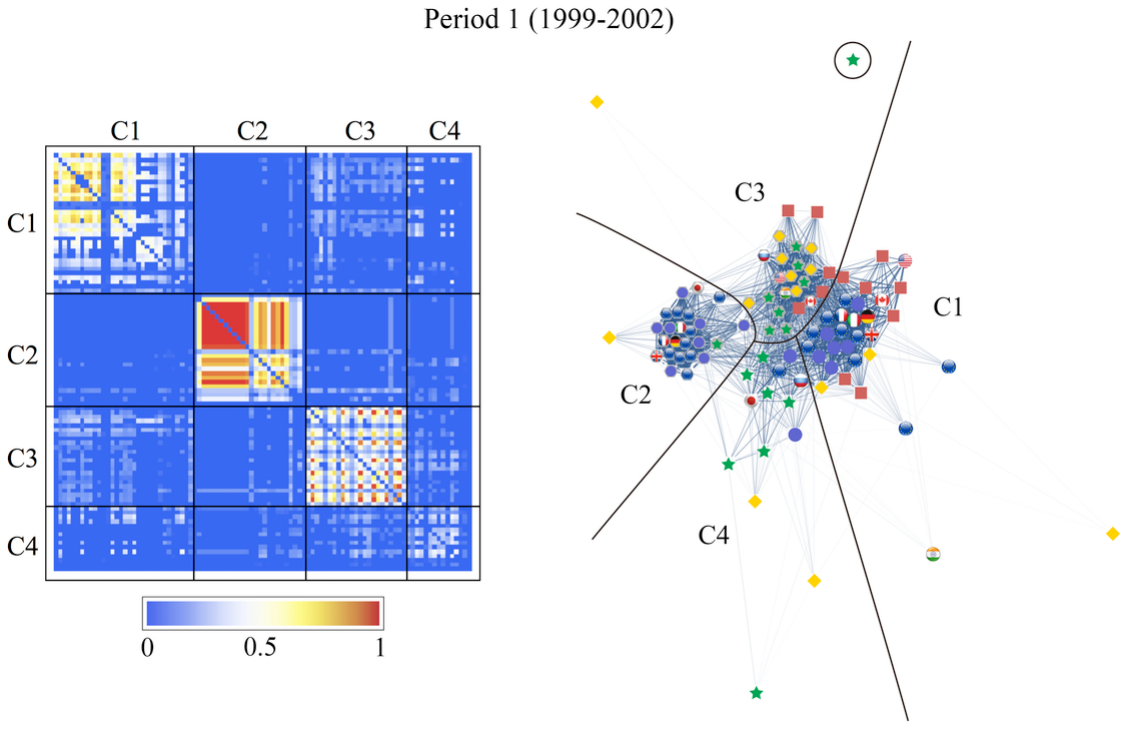
Same as Fig 13, for period 1 (1999–2002). The network consists of four communities as it does in the entire period. The major equity community, C1, has the lowest strength of synchronization in this period while one of the two currency communities, C2, is relatively isolated from the rest of the network; in contrast, the other currency community, C3, is closely related to C1; the Asian equity community C4 is not so strongly connected.

**Fig 15:**
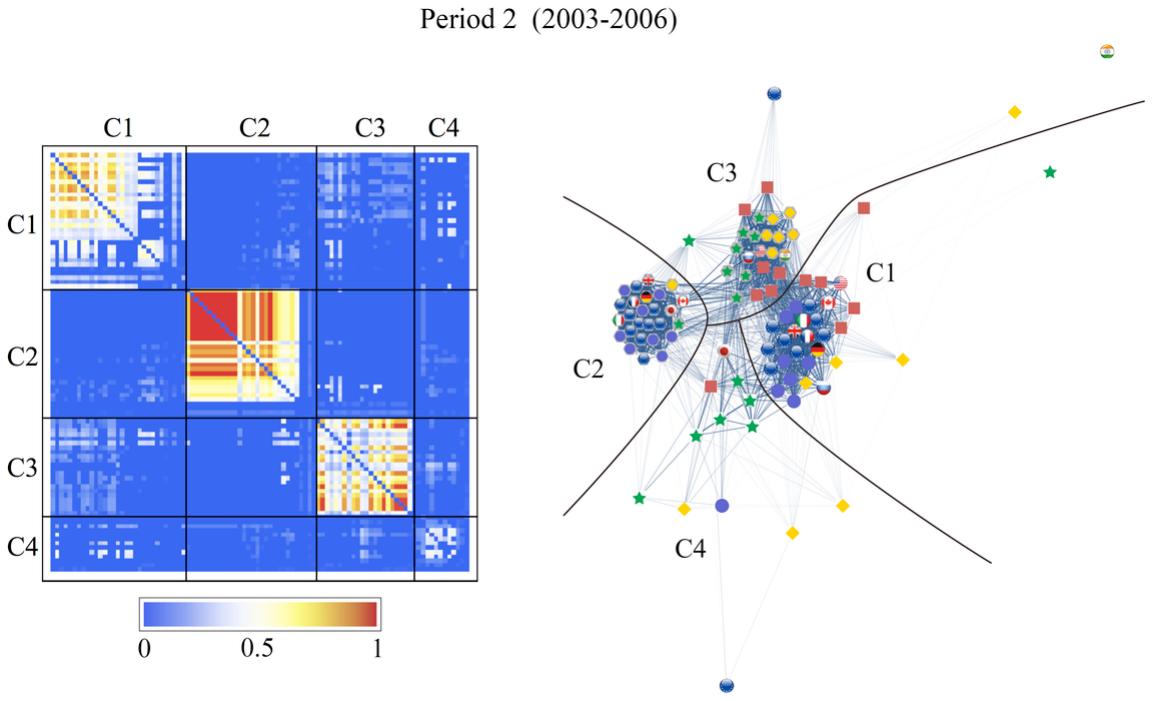
Same as Fig 13, for period 2 (2003–2006). The network is decomposed into four communities to the largest extent in this period; we especially observe that the formation of community C2 is very tight.

**Fig 16:**
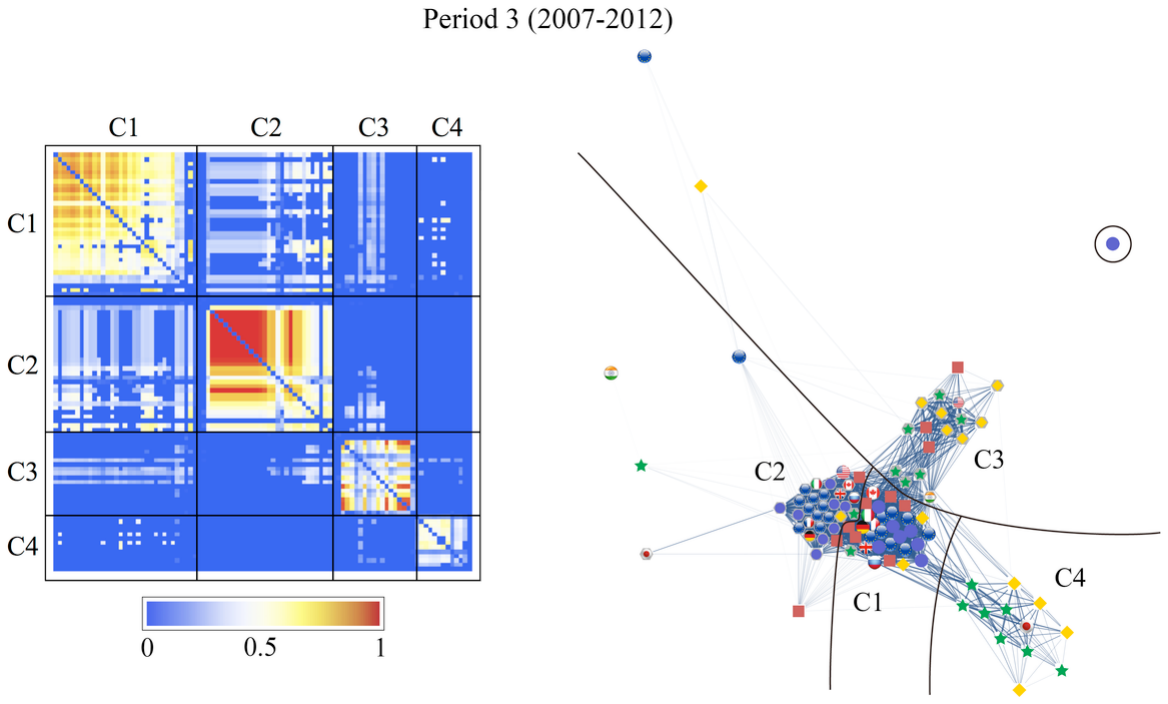
Same as Fig 13, for period 3 (2007–2012). The community structure observed here is quite similar to that obtained in the entire period. This indicates that the global crisis brings a profound influence on the global financial network. Community C1 has the highest degree of synchronization in this period; C2 is now strongly connected to C1 while C3 is further apart from C1 compared to previous sub-periods; C4 has been well established as a group of synchronizing nodes.

We depict the community detection results as an adjacency matrix on the left-hand side and a graphical representation of the networks on the right-hand side of Figs 13–16. The components, given by *r*_*αβ*_, of the adjacency matrix take values between 0 (no synchronization) and 1 (perfect synchronization). The adjacency matrix is visualized using a color code based on a temperature map scheme in which the color changes continuously from blue (*r*_*αβ*_ = 0) to red (*r*_*αβ*_ = 1) through white (*r*_*αβ*_ = 0.5). The community structure does not change significantly if the network is constructed with a larger or smaller cutoff for the phase differences, e.g., *θ*_*c*_/*π* = 0.15 or 0.05 (see Text D in S1 File). However the nature of the communities and the interrelationships between them display different characteristics from period to period.

The visualized adjacency matrices indicate that community C1 increases its synchronization between periods 1 and 3, especially among the European stock markets. This is confirmed by calculating the average correlation coefficients within the communities as shown in Table 2, where we observe an increase from 0.25 in period 1 to 0.57 in period 3. The nodes in C2, consisting mainly of the European currencies, are most tightly coupled in period 2. The average correlation coefficient for C2 increases from 0.58 to 0.72 from period 1 to period 2, and consequently decreases to 0.48 in period 3. Community C3, led by the US dollar, holds approximately the same synchronization strength throughout the entire period with an average correlation coefficient of 0.35. The community C4, dominated by an Asian stock markets group, is well established with an average correlation coefficient increasing from 0.10 in period 2 to 0.31 in period.

Both the adjacency matrices and the network layouts show that C2 is independent in periods 1 and 2 but suddenly changes its characteristics in period 3 when it is strongly connected to C1. On the other hand, C3 is closely related to C1 in the first two periods, and exhibits a stronger connection with C2 in the last period. The independence of community C4 from the rest of the network steadily increases. These findings receive confirmation in the results for the average correlation coefficients across the communities listed in Table 2.

**Table 2:** Magnitude of the complex correlation coefficients averaged over all pairs of nodes within each of the four communities, C1, C2, C3, and C4, and across the communities for the entire period (1999–2012) and the three sub-periods, period 1 (1999–2002), period 2 (2003–2006), and period 3 (2007–2012). These results measure how tightly the communities are synchronized and to what extent residual coupling still remains across the communities.

Community Pair	Entire Period	Period 1	Period 2	Period 3
C1-C1	0.406	0.246	0.307	0.566
C2-C2	0.621	0.576	0.724	0.482
C3-C3	0.265	0.351	0.371	0.347
C4-C4	0.166	0.127	0.101	0.312
C1-C2	0.094	0.005	0.007	0.158
C1-C3	0.053	0.051	0.039	0.051
C1-C4	0.021	0.034	0.024	0.021
C2-C3	0.030	0.017	0.012	0.022
C2-C4	0.003	0.019	0.010	0.000
C3-C4	0.018	0.038	0.016	0.009

We note that the US stock market and the yen occupy relatively peripheral positions in the financial network throughout the entire period. We also find that the Indian and Korean stock markets do not belong to C4 and remain largely isolated from the main body of the network irrespective of time period.

Fig 11 shows how the community structure of the stock and foreign exchange co-moving synchronization network is approximately stable over the entire period and during the three sub-periods, i.e., Period 1, 1999–2002 (mild crisis), Period 2, 2003–2006 (relatively calm), and Period 3, 2007–2012 (severe crisis).

#### Temporal relationships between communities

According to the definition of ${\tilde{C}}_{\alpha \beta }$, the value of *θ*_*αβ*_ is the lead phase angle of *β* against *α*. We have already identified four communities arising from the coherent motion of nodes in the equity-currency network. We thus expect that some of them may have a definite lead-lag relation to each other.

**Fig 17:**
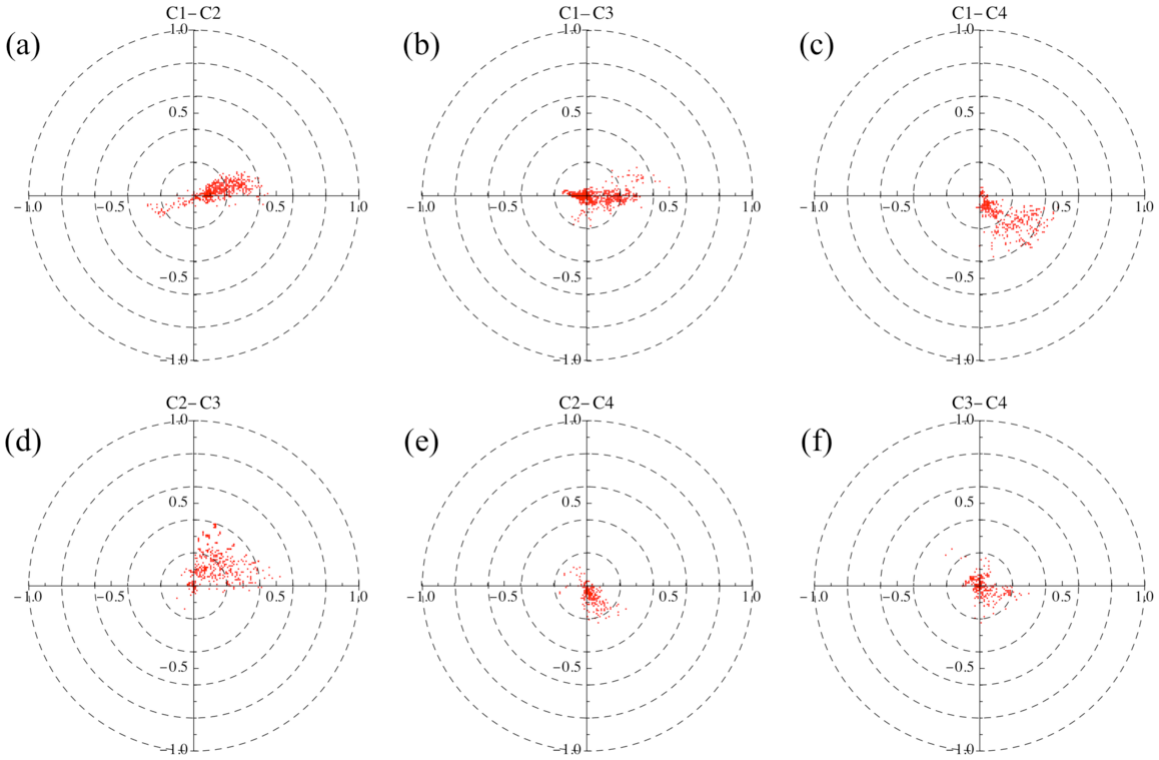
Scatter plots of the complex correlation coefficients across the four communities on the complex plane. (a) for C1-C2, (b) for C1-C3, (c) for C1-C4, (d) for C2-C3, (e) for C2-C4, and (f) for C3-C4. If the correlation coefficients between C*m* and C*n* have a positive median in regards to the distribution of their phases weighted by the associated magnitudes, we infer that C*n* leads C*m*; if the median is negative, then we infer that C*m* leads C*n*.

Fig 17 shows the correlation coefficients calculated between different communities for the entire period on a complex plane. Note that the distribution of their phases is significantly polarized for every pair of communities. To quantify such a lead-lag relation between C*m* and C*n*, we compute the median for a weighted distribution of the phase differences between the two communities. We define the distribution *ρ*_*mn*_(*x*) in terms of Dirac’s *δ* function *δ*(*x*) as

$$\begin{array}{c} \rho_{mn}\left(x\right)=\sum_{\alpha \in Cm,\;\beta \in Cn}r_{\alpha \beta }\;\delta \left(x-\theta_{\alpha \beta }\right)\;/\sum_{\alpha \in Cm,\;\beta \in Cn}r_{\alpha \beta }\;, \end{array}$$ (28)

where the correlation strength is understood in terms of weight. The results are 0.053 for C1-C2, −0.010 for C1-C3, −0.205 for C1-C4, 0.276 for C2-C3, −0.318 for C2-C4, and −0.045 for C3-C4 in units of 1/*π*.

**Fig 18:**
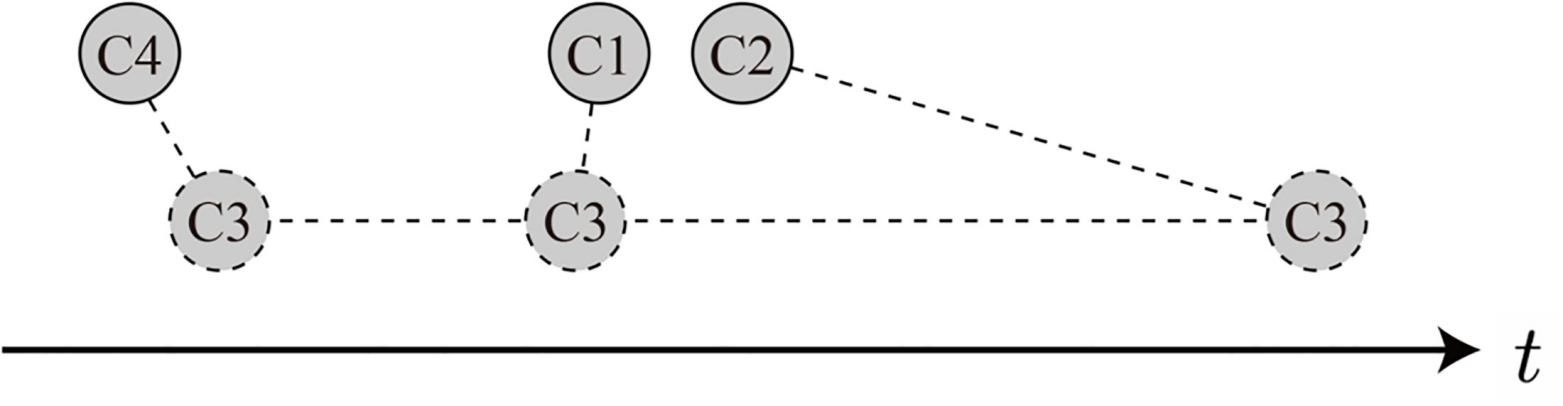
Lead-lag diagram for the four communities inferred from the results in Fig 17. The three communities, C1, C2, and C4, are steadily aligned in the time direction (C2 leading C1 and C4), while C3 has three possible positions depending on which binary correlation is emphasized, C1-C3, C2-C3, or C3-C4.

Fig 18 shows that community 2 usually leads community 1 which in turn leads community 4, and that community 3 does not have a stable position with respect to the other communities. The distances between communities represent the average phase differences *θ*_*αβ*_ weighted by the magnitudes *r*_*αβ*_.

We begin by determining the triangular relationship among C1, C2, and C4 by minimizing the geometric discrepancy involved in their phase differences. We set the discrepancy *D* as

$$\begin{array}{c} D={\left(\Theta -\theta_{12}\right)}^{2}+{\left(\Phi -\theta_{14}\right)}^{2}+{\left(\Psi -\theta_{24}\right)}^{2}, \end{array}$$ (29)

where Θ, Φ, and Ψ are variables corresponding to *θ*_12_, *θ*_14_, and *θ*_24_ to be optimized with constraint Θ − Φ + Ψ = 0. The geometrically consistent phase differences thus obtained are compatible with the original ones with absolute errors of less than 0.02*π* (3.6 degrees). This indicates the three communities are interrelated with a solid causal relation given by C2 → C1 → C4. Note that the relation holds over both the entire period and in period 3, i.e., the community structure average for the entire period is very similar to period 3.

Although the C1-C2-C4 triangle is well established, the lead-lag relations determined by the pairs of C1-C3, C2-C3, and C3-C4 give C3 three positions that significantly differ from each other (see Fig 18). The phase difference between two communities only has meaning on average. This is also the case for the notion of “community of synchronizing nodes.” If the three lead-lag relations indicated in the C1-C3, C2-C3, C3-C4 diagrams are magnified alternatively in time, the multiphase behavior of C3 toward the triangular relationship among C1, C2, and C4 appears.

The worldwide financial networks thus turned out to have communities of synchronizing nodes with rich lead-lag effects. Suppose that we carried out the same community analysis by adopting PCA based on a real correlation matrix instead of CHPCA based on a complex one. We would still be able to identify groups of nodes moving in phase as forming communities. However, these PCA based communities would be simply regarded as being either weakly coupled with each other or moving out of phase by 180 degrees, depending on the degree of correlation between them.

## Conclusion

In this paper we study the interactions between equity and foreign exchange markets for 48 countries between 1999 and 2012. We use insights from statistical physics and network science to model relationships and influences between the foreign exchange and stock markets as two interrelated networks that have corresponding nodes and bi-directional dependencies. We use Complex Hilbert Principal Component Analysis (CHPCA) approach to build an interdependent network model for studying the dynamics of this coupled financial system. We construct complex time series by using the Hilbert transformation of the time series for the imaginary component. Using Rotational Random Shuffling (RRS) we find a relatively small number of significant eigenmodes (5 or 6 out of 96 total eigenmodes) and investigate the results obtained by them. We closely examine the eigenvector components corresponding to the three largest eigenvalues of the complex correlation matrix and determine the importance of smaller but significant eigenmodes.

Being able to extract important information about lead-lag relationships between global financial markets and currencies, ridding the results from the noise in the data, can be used to better understand and monitor signs of possible risk contagion based on the values of significant eigenmodes and the largest values of the eigenvectors associated with the important eigenvalues. For instance, in the 6th eigenmode, the dominant contributions come from the Iceland’s stock market, followed by the Icelandic krona which reveals instability at a particular time (October 2008) when the Icelandic banking crisis started, and Island’s stock market lost over 90 percent of its value, while the Icelandic krona was significantly devalued.

For the entire period between 1999–2012, we observe that in general currency appreciations lead or contribute to positive equity market returns. We also find that the US equity market is an indicator of the future performance of other global stock markets. We also study three distinct sub-periods, the “mild crisis” (1999–2002), “calm” (2003–2006), and “severe crisis” (2007–2012) periods, and find that under different macroeconomic conditions the interactions between the foreign exchange and stock markets vary. We observe that during the mild crisis (1999–2002) sub-period, global equity markets were most able to forecast currency returns. During the calm (2003–2006) sub-period, stock markets exhibited weaker correlations than the foreign exchange markets, which appeared to be more correlated. During the severe crisis (2007–2012) sub-period the interactions between the forex and equity markets were very strong, underlining this period’s strong influence on the overall relationship between currency and stock markets.

By dividing the analysis into distinct (mild crisis, clam, and severe crisis) periods, we take in consideration the different economic dynamics, where in tranquil periods economic variables are sound and stable, compared to crisis periods, when economic variables are volatile and go through an adjustment process [69]. Our CHPCA analysis can be used as an early warning system (EWS) for systemic risk contagion based on the resulting lead-lag relationships as obtained by global financial markets and foreign exchange network analysis. For example, if a distress originates in specific financial market, other markets could be affected adversely if lagging closely behind the distressed market. This information, in real time, could be very useful for policy makers to react promptly to prevent cascading losses in closely related economies. Liberalization of financial markets and rapid innovation have increased the need for EWS considering policy maker’s objective to prevent crisis spillovers that can be detrimental and long-lasting [70].

We study the intra-relations (within one market) as well as inter-relations (between the two markets) for the forex and equity networks and find distinct clustering in the network that persists for the entire period, which is characterized by behavior that is similar to that in the three distinct sub-periods. We identify four major communities that are approximately stable over time and do not change when the cutoff of the phase differences is changed. The first community (C1) is comprised of equity markets dominated by Europe, the USA, South America, and Canada, the second (C2) of the Canadian dollar and mainly European currencies including the Euro, the third (C3) of the Russian ruble, the US dollar, and selected South American, Asian, and Middle Eastern currencies, and the fourth (C4) of Asian equity markets, including Japan, and the Middle East.

We propose the community structure framework to assess financial system risk and the distance among markets, based on the communities to which markets and currencies belong. This could infer lower or higher vulnerability of the markets and currencies based on weaker or stronger relationships obtained by the CHPCA algorithm. Our method builds on coincidental financial market measures (returns) and forward looking indicators (lead-lag relationships) that could reveal likelihood of simultaneous loss in value in different financial markets or devaluations of currencies. Significant tight clustering of financial markets and currencies in synchronization network communities can serve as warning signal for macro-prudential policy action.

We study the lead-lag relationships between the four communities and find a solid causal relationship for C2 leading C1 and C4, and C1 leading C4, while C3’s position in the lead-lag diagram is not settled, but takes three different positions that significantly differ from each another. The network-based approach to model the dynamic structure of equity and foreign exchange markets allows us to capture the topology and interdependence of the two global markets and to find their lead-lag relationships for different macroeconomic environments.

Understanding different stages in the economic cycle, and assessing the period that prevails is very important for sound monitoring, regulating, and prompt preventative actions.

The main contribution of our model is that CHPCA combined with RRS offers superior approach for discovering multi-dimensional intrinsic relations within the global financial network of currencies and financial markets. Our approach offers three methodological advantages compared to previous studies: (i) detecting beyond-pairwise lead-lag relationships compared to traditional Granger causality and cross-correlation analysis; (ii) extracting dynamical correlations simultaneously; and (iii) using RRS to provide a sound null hypothesis for identifying statistically significant correlations without making any distribution assumptions of the empirical financial time series that we study.

Another important aspect of our study is the global analysis of “economic regions” rather than “geographical regions.” These economic regions we can see emerge according to the composition of our network communities. For example, American stock markets belong to the same community (C1), but American currencies belong to the same community (C3) as Asian currencies. The Japanese Yen does not belong to the community dominated by Asian currencies but to the community dominated by European currencies (C2), where we also find the Australian Dollar and the South African Rand. Thus we can emphasize that an “economic region” is in fact quite different from a “geographic region” and that studies limited to geographic regions will not detect the interrelationships among truly global financial and economic trends.

The community classification of currencies and financial markets reveal similar features when they belong to the same communities. Markets and currencies with similar characteristics (being part of the same community) are prone to affect each other to a greater extent. In this study we reveal the structure of the coupled financial network as basis for closely monitoring relationships among global financial markets and currencies to observe temporal structural changes. These observed structural changes could be interpreted as early warning signals for systemic risk buildup and possible contagion.

The model that we propose in this manuscript could be expanded beyond our study of the interdependencies between the foreign exchange market and the stock market to include global fixed income, credit default swaps, interest rates, options, futures, and other financial markets and instruments. We suggest that this methodology could be useful in the development of a tool for real-time monitoring of the dynamics of global financial markets, one that could enable policy makers and regulators to daily monitor systemic risk fluctuations in the global financial system.

## Supporting Information

S1 File Summary statistics, details of methodologies, and other supplementary materials. Summary statistics of the time-series (Text A); Detailed study of the CHPCA eigensystem for each period (Text B); Visualization of significant and complexified time-series (Text C); Dependence of the community structures on the cutoff angle (Text D).
(PDF)
