# Supplementary material for: Interdependencies and Causalities in Coupled Financial Networks
Source: PLoS One. 2016 Mar 15;11(3):e0150994. doi: 10.1371/journal.pone.0150994 (PMC4792465; doi:10.1371/journal.pone.0150994)
Supplement: S1 File — Summary statistics of the time-series (Text A); Detailed study of the CHPCA eigensystem for each period (Text B); Visualization of significant and complexified time-series (Text C); Dependence of the community structures on the cutoff angle (Text D). (PDF) [file pone.0150994.s001.pdf]

Supporting Information: Summary statistics, details of methodologies, and other supplementary materials

Irena Vodenska[1,2,*], Hideaki Aoyama[3], Yoshi Fujiwara[4], Hiroshi Iyetomi[5], Yuta Arai[5]

1 Metropolitan College, Boston University, 808 Commonwealth Avenue, Boston, MA 02215, USA
2 Center for Polymer Studies, Boston University, 590 Commonwealth Avenue, Boston, MA 02215, USA
3 Graduate School of Sciences, Kyoto University, Kyoto 606-8502, Japan
4 Graduate School of Simulation Studies, University of Hyogo, Kobe 650-0047, Japan
5 Department of Mathematics, Niigata University, Niigata 950-2181, Japan

*** vodenska@bu.edu**

A Summary statistics of the time-series

Table A1 displays an overview of the summary statistics for each country's stock market performance and currency returns. Note that while Pakistan, Russia, and Venezuela show daily stock market returns of over 0.079%, 0.095%, and 0.12%, respectively, European countries such as Greece, Italy, and Portugal show negative daily stock market returns of over −0.03%, −0.02%, and −0.018%, respectively, between 1999 and 2012. Almost all of the American, Asian, and the Middle Eastern stock markets show positive returns, with the exception of Japan with a −0.008% return. Russia, Iceland, and Argentina show the highest stock market volatilites, while Malta, Malaysia, and Mauritius appear to have the lowest. Iceland exhibits the largest positive skew, significantly different from all the other countries, and the highest kurtosis, followed by Mauritius showing the next highest stock market kurtosis, approximately 10 times lower than Iceland. European and Asian countries show mixed currency returns, while the American and the Middle Eastern countries show primarily negative currency returns. Currencies display lower volatilites than stock markets. Venezuela and Argentina exhibit the largest positive skews and kurtosis, with the magnitude for Venezuela being approximately three times the magnitude for Argentina for both skewness and kurtosis.

Table A2 displays the summary statistics for periods 1, 2, and 3. In period 2 (2003–2006) we see the highest positive global stock market returns of 0.09−0.13% and a relatively low volatility of 0.01−0.013%. The currency results for the same period are mixed. The stock markets on average are not significantly skewed. The Middle Eastern stock markets show the highest kurtosis in period 2, while the European stock markets exhibit highest kurtosis in period 3. Currencies on average show mixed returns with relatively low volatility. American currencies exhibit positive skewness and significant kurtosis in all 3 periods.

Table A1. Summary Statistics for the log−returns. Here μ is the mean in units of 10^{-5}, σ the standard deviation in units of 10^{-3}, γ_1 the skewness (the third standardized moment), and β_2 the kurtosis (the fourth standardized moment, equal to three for normal distribution).

		Stock Index				Currency			
No.	Country	μ	σ	γ_1	β_2	μ	σ	γ_1	β_2
1	UK	0.03	5.50	−0.14	8.79	−1.30	2.45	−0.21	5.56
2	Austria	9.06	6.34	−0.31	10.64	0.39	2.70	0.09	4.31
3	Belgium	−4.17	5.73	0.06	8.79	0.39	2.70	0.09	4.31
4	Finland	4.71	7.01	−0.07	5.92	0.39	2.70	0.09	4.31
5	France	−0.95	6.65	0.03	7.66	0.39	2.70	0.09	4.31
6	Germany	4.99	6.89	0.00	7.18	0.39	2.70	0.09	4.31
7	Ireland	−4.59	6.22	−0.56	10.66	0.39	2.70	0.09	4.31
8	Italy	−9.25	6.63	−0.05	7.82	0.39	2.70	0.09	4.31
9	Netherlands	−5.37	6.63	−0.09	8.92	0.39	2.70	0.09	4.31
10	Portugal	−7.91	5.04	−0.14	10.63	0.39	2.70	0.09	4.31
11	Spain	−2.21	6.65	0.11	8.15	0.39	2.70	0.09	4.31
12	Greece	−13.13	7.80	0.03	7.09	−0.07	2.67	0.10	4.37
13	Malta	11.60	3.59	1.35	18.92	0.72	2.65	0.20	18.54
14	Slovakia	8.51	5.45	−1.08	19.05	4.70	2.80	0.13	4.36
15	Norway	16.59	7.05	−0.55	9.33	2.65	3.23	−0.13	6.17
16	Sweden	5.41	6.95	0.07	6.10	1.60	3.32	0.02	5.34
17	Iceland	−5.16	9.10	−37.38	1838.72	−8.31	4.32	0.54	65.35
18	Switzerland	−0.58	5.32	−0.03	9.24	3.80	3.00	−0.52	11.96
19	Czech	11.52	6.45	−0.44	14.70	4.44	3.38	0.05	5.45
20	Denmark	9.71	5.66	−0.22	8.61	0.41	2.70	0.09	4.31
21	Hungary	12.59	7.16	−0.04	8.94	−1.34	3.97	−0.39	7.37
22	Poland	14.04	5.97	−0.36	6.62	0.44	3.81	−0.15	7.14
23	Russia	41.42	10.45	−0.03	13.82	−5.72	2.43	−1.31	38.95
24	USA	1.77	5.70	−0.15	10.48	−1.04	1.34	−0.08	6.04
25	Canada	7.74	5.16	−0.64	11.67	4.15	2.69	−0.12	4.97
26	Mexico	28.56	6.26	0.10	7.32	−4.14	3.14	−0.64	11.72
27	Brazil	26.12	8.46	0.76	19.30	−7.34	4.97	−0.23	14.26
28	Argentina	22.51	9.06	−0.08	8.52	−19.98	4.11	−14.14	468.30
29	Chile	20.21	4.55	0.08	11.70	−1.18	2.87	−0.18	6.08
30	Peru	32.56	6.19	−0.52	14.99	1.50	1.74	−0.04	7.56
31	Venezuela	54.59	6.38	−0.29	25.79	−25.18	6.41	−30.25	1420.36
32	India	13.74	6.29	−0.50	9.85	−4.07	1.76	0.32	7.28
33	Sri Lanka	26.71	5.19	0.36	33.00	−8.46	1.92	−2.52	58.49
34	Indonesia	28.35	6.49	−0.36	9.57	−3.18	3.98	0.27	16.24
35	Japan	−3.41	6.52	−0.39	10.17	2.17	2.94	0.03	7.02
36	South Korea	8.24	7.03	−0.49	9.85	0.43	3.20	0.09	27.03
37	Malaysia	12.59	4.14	−0.52	11.95	1.54	1.55	−0.11	6.36
38	Thailand	16.23	6.44	−0.36	12.04	1.01	1.70	−0.12	8.22
39	Philippine	12.88	5.83	0.40	18.48	−1.68	2.16	3.34	75.49
40	Hong Kong	9.67	6.90	−0.05	10.38	−1.05	1.32	−0.05	6.08
41	Australia	6.39	4.41	−0.50	9.16	5.29	3.66	−0.25	12.06
42	Israel	15.84	5.66	−0.26	6.10	0.17	2.19	−0.12	7.86
43	Pakistan	34.31	6.37	−0.25	6.48	−9.03	2.14	−0.10	12.50
44	Saudi Arabia	18.58	6.60	−1.04	13.6	−1.05	1.36	−0.06	5.93
45	South Africa	24.43	5.94	−0.11	6.56	−5.35	4.82	−1.08	16.79
46	Oman	10.69	4.36	−0.54	20.96	−1.04	1.39	0.00	6.62
47	Qatar	21.59	7.96	−0.25	23.25	−1.06	1.34	−0.07	6.09
48	Mauritius	15.63	3.67	−0.46	191.26	−3.56	3.72	−0.36	12.03

Table A2. Summary Statistics for the log-returns in each period. Here μ is the mean in units of 10^{-5}, σ the standard deviation in units of 10^{-3}, γ_1 the skewness, and β_2 the kurtosis. A bar above a symbol indicates that it is the average over all the countries in each geographic region, where "Europe" contains countries #1–#23, "America" #24–#31, "Asia" #32–#41, and "Middle East" #42–#48 in Fig 1.

	Stock Index				Currency			
Region	$\bar{\mu}$	$\bar{\sigma}$	$\bar{\gamma_1}$	$\bar{\beta_2}$	$\bar{\mu}$	$\bar{\sigma}$	$\bar{\gamma_1}$	$\bar{\beta_2}$
Period 1 (1999–2002)								
Europe	−3.72	6.48	0.03	6.41	−2.48	2.82	−0.09	8.36
America	8.46	6.94	0.43	9.96	−18.25	3.54	−1.89	41.12
Asia	−2.57	6.47	0.57	15.84	−3.47	2.51	−0.03	25.08
Middle East	15.02	5.51	0.17	12.20	−2.87	2.23	−0.18	7.66
Period 2 (2003–2006)								
Europe	38.89	4.48	−0.23	7.4	5.11	2.63	0.07	4.23
America	54.41	4.88	−0.08	6.02	0.02	2.73	−0.42	31.08
Asia	33.22	4.81	−0.52	10.77	−0.42	1.8	−0.02	5.05
Middle East	46.01	5.58	−0.46	44.78	−4.20	2.17	−0.17	4.88
Period 3 (2007–2012)								
Europe	−13.61	7.46	−1.37	50.53	−1.16	3.17	−0.01	7.96
America	14.68	6.91	−0.52	15.13	−3.38	3.49	−4.84	190.62
Asia	10.22	6.06	−0.53	11.34	0.72	2.61	−0.10	10.56
Middle East	6.35	5.79	−0.41	11.17	−2.26	2.65	−0.33	10.59

B Detailed study of the CHPCA eigensystem for each period

In this appendix, we give the detailed account of the CHPCA eigenvalues and eigenvectors for each of the three periods.

The comparisons of the eigenvalues and the RRS eigenvalue distribution are given in Fig. B1 for periods 1, 2 and 3 from top to the bottom in the manner of Fig. 2. From these, we learn that top 5 eigenvalues for periods 1 and 2, and top 6 eigenvalues for period 3 are clearly outside of the range of the RRS distribution and are significant.

These eigenvectors of the three periods may be decomposed in terms of the eigenvectors for the whole period as follows:

$$\boldsymbol{V}_p^{(n)} = \sum_{m=1}^{N} c_p^{(n,m)} \boldsymbol{V}^{(m)}, \tag{B.1}$$

where $\boldsymbol{V}_p^{(n)}$ is the n-th eigenvector for the period $p\ (= 1, 2, 3)$. Since $\boldsymbol{V}$ and $\boldsymbol{V}_p$'s span complete set, the decomposition coefficients $c_p^{(n,m)}$ satisfies

$$\sum_{m=1}^{N} \left| c_p^{(n,m)} \right|^2 = 1. \tag{B.2}$$

The coefficients $\left| c_p^{(n,m)} \right|^2$ are given in Fig. B2, with m on the x-axis and n in descending

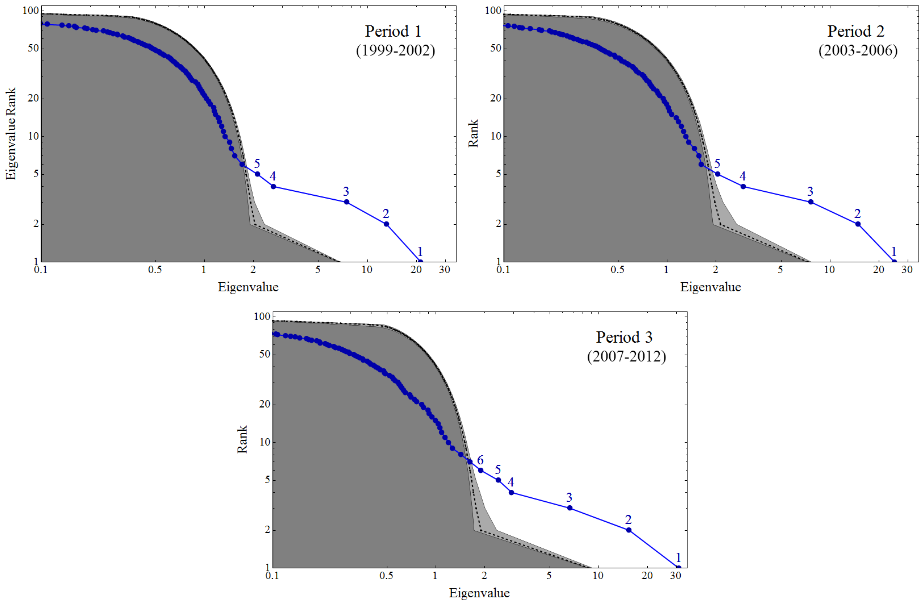

Figure B1. Significant eigenvalues identified by the CHPCA with RRS results for periods 1-3. The blue dot denoted 'n' shows the n-th largest CHPCA eigenvalue (x-axis) and the CHPCA eigenvalue rank (y-axis). The gray small dots and the lighter gray area show the average RRS and the 99% range. The largest 5 eigenvalues in periods 1 and 2, and the largest 6 eigenvalues for period 3 are clearly outside of each of their RRS ranges. and show significant relationships in the interdependent network.

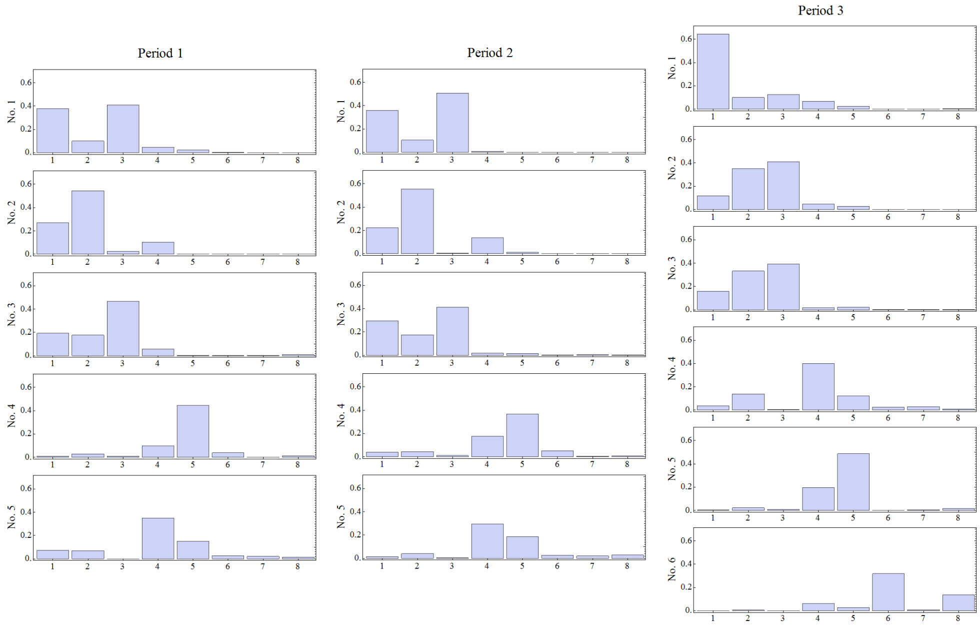

Figure B2. Decomposition of the eigenvectors in each period in terms of eigenvectors of the entire period.

order from top to bottom in each plot (denoted as "No.n"). We observe the following in these plots:

1. Top 5 (for periods 1 and 2) and 6 (for period 3) eigenvectors are well-approximated by linear-combinations of the top 6 (significant) eigenvectors of the whole period. This confirms that 6 significant eigenmodes of the whole period are sufficient for looking into specific periods, validating mode-signal analysis by the top 6 eigenmodes, described in the section C.

2. Among all three periods, the eigenvector structure of period 3 most similar to the structure of the entire period: In the period 3, No.1 eigenvector is mostly represented by the top eigenvector of the entire period, No.2 and No.3 are mixtures of the second and the third eigenvectors of the entire period, while No. 4, 5, and 6 are mostly based on their corresponding counterpart of the entire period. This confirms the fact that period 3, representing the severe crisis period and exhibiting significant co-motion within the synchronization network, dominates the entire period and plays essential part in the determination of the eigenmodes of the entire period.

C Visualization of significant and complexified time-series

In our analysis of complex correlation matrix, the complexified time-series are expanded in terms of the eigenvectors for the complex correlation matrix, namely in the expansion given by Eq. (15). We found by the RRS method that the number of significant eigenvalues and corresponding eigenvectors can be estimated to be 6 for the entire period.

We are able to select those first $N_s = 6$ significant terms in the full expansion of N terms:

$$\tilde{w}_\alpha^{(N_s)}(t) := \sum_{n=1}^{N_s} a^{(n)}(t)\, \boldsymbol{V}_\alpha^{(n)}, \tag{C.1}$$

regarding the other terms as "noise". Fig. C1 depicts the absolute values of $a^{(n)}(t)$ for $n = 1, 2, \ldots, N_s = 6$ (from top to bottom) during the entire period of time.

One can observe that on average, the first mode-signal corresponding to the largest eigenvalue dominates over the other mode-signals. Recall the relation Eq. (16) which states that the *average* of $a^{(1)}$ is greater than that of $a^{(n)}$ for $1 < n$, because $\lambda^{(1)} > \lambda^{(n)}$. The plots in Fig. C1 show that the first mode-signal dominates the others, not only in an averaged sense but also for almost all time. Similar observation holds for the other mode-signals in decreasing order of n. The striking exception is the $n = 6$ mode-signal, which becomes as strong as the $n = 1$ at the time of the Lehman Brothers crisis, as discussed in the subsection of the paper, "Insights from smaller significant eigenmodes".

We can further convert from $\tilde{w}_\alpha(t)$ to $\tilde{r}_\alpha(t)$ by using Eq. (8) in the opposite way:

$$\tilde{r}_\alpha^{(N_s)}(t) := \langle \tilde{r}_\alpha \rangle_t + \sigma_\alpha \cdot \tilde{w}_\alpha^{(N_s)}(t) \tag{C.2}$$

so that we can construct the significant and complexified time-series by using those 6 mode-signals and corresponding eigenvectors.

The resulting time-series $\tilde{r}_\alpha^{(N_s)}(t)$ are significant co-movements that can be visualized by a set of points, equities or currencies of α, on the complex plane at each point of time t. We provide as a supplementary material a visualization for the movements of those points from January 1999 to December 2012. Users can manipulate the visualized co-movements of equities and currencies along the time-line to see how significant co-movements change at epochs; mild crisis, calm period, severe crisis, and particular dates of interest.

How the points of equities and currencies are dispersed on the complex plane can be quantified by a measure of dispersion at each time t:

$$D(t) := \sqrt{\sum_\alpha |\tilde{r}_\alpha^{(N_s)}(t)|^2} \tag{C.3}$$

The temporal change of $D(t)$ is shown in Fig. C2. We can observe many bursts of $D(t)$ at different times, which signal significant changes at the respective periods. If, in addition, a set of points in the complex plane has a radial line-up, such a set of points implies a significant co-movements. It should be noted in Fig. C2 that the most striking dispersion occurs during the Lehman Brothers crisis in the third quarter of 2008, which brought about a subsequent volatile period continuing into the European sovereign debt crisis that followed.

See [1] for additional results.

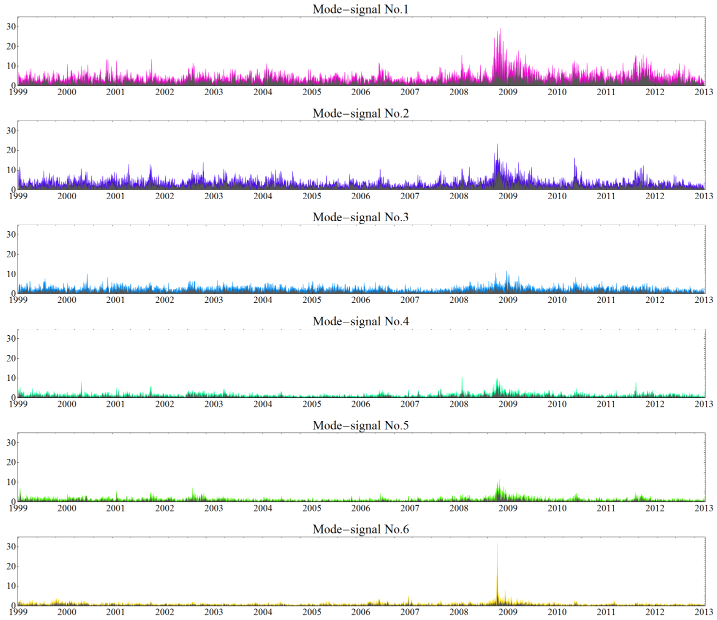

Figure C1. Absolute values of the mode-signals $a^{(n)}(t)$ for $n = 1, 2, \ldots, N_s = 6$ during the entire period of time. Mode-signal $a^{(n)}$ dominates the others $a^{(m)}$ for $m > n$, not only in the average sense (as expected from the relation in Eq. (16), that is satisfied by the mode-signals), but also for all the periods. A striking exception is the $n = 6$ mode-signal, which becomes as strong as the first, $n = 1$, mode-signal at the time of the Lehman Brothers crisis. See also subsection **Complex Correlation Matrix** in the main body of the paper.

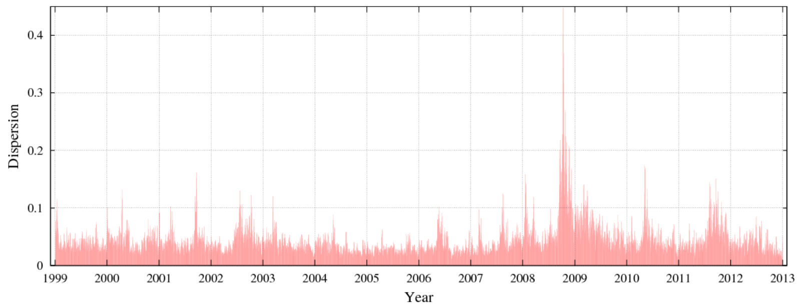

Figure C2. Dispersion $D(t)$ of the equities and currencies, defined by Eq. (C.3), calculated from the significant and complexified time-series, $\tilde{r}_\alpha^{(N_s)}(t)$. We observe many bursts with significant signal changes at different time periods. If, in addition to the dispersion, a set of points in the complex plane has a radial line-up, this implies significant co-movements. The most striking dispersion corresponds to the Lehman Brothers crisis in the third quarter of 2008 followed by a subsequent volatile period corresponding to the European sovereign debt crisis.

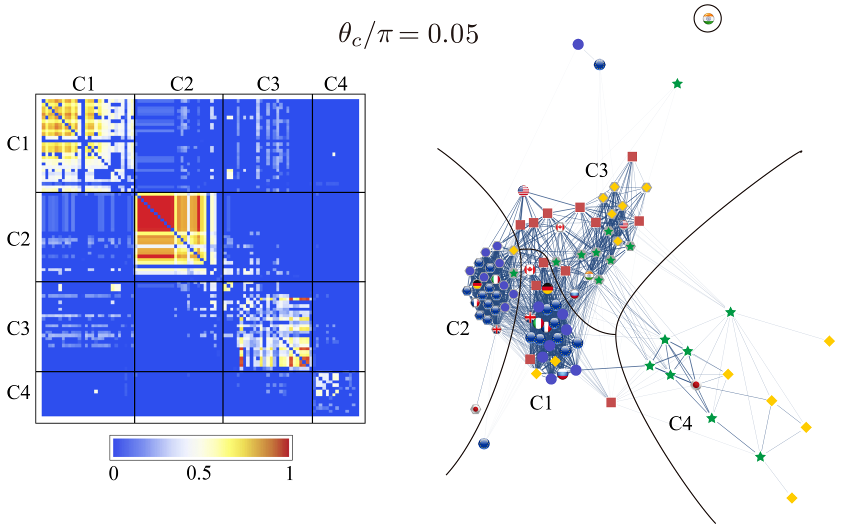

Figure D1. Community structure for $\theta_c = 0.05$. Lowering θ_c by half decreases the number of links from 3,890 to 2,616 and hence makes the network considerably sparser. However, the four community structure resembling to that in Fig. 13 still survives.

D Dependence of the community structures on the cutoff angle

The choice of the cutoff angle θ_c to determine synchronizing nodes (markets and currencies) needs careful study. Here we demonstrate to what extent the community structure depends on θ_c in Figs. D1 and D2 corresponding to Fig. 13 for the entire period, where the cutoff value one and a half times or half as large as that in Fig. 13 is adopted. The number of links drops from 4,644 for $\theta_c/\pi = 0.15$ to 3,890 for $\theta_c/\pi = 0.1$ and to 2,616 for $\theta_c/\pi = 0.05$. We see that the four community structure as has been already identified is stable against such substantial change of θ_c. This is also true for all of the results obtained with the varied cutoffs in the three partial periods with two exceptions. In periods 1 and 2, the two stock market communities are combined into one group at the largest value of θ_c. However, one can easily identify the two sub-communities in the combined community. Therefore, we infer that the construction of the synchronization network and its community structure is robust with respect to the choice of the cutoff value θ_c.

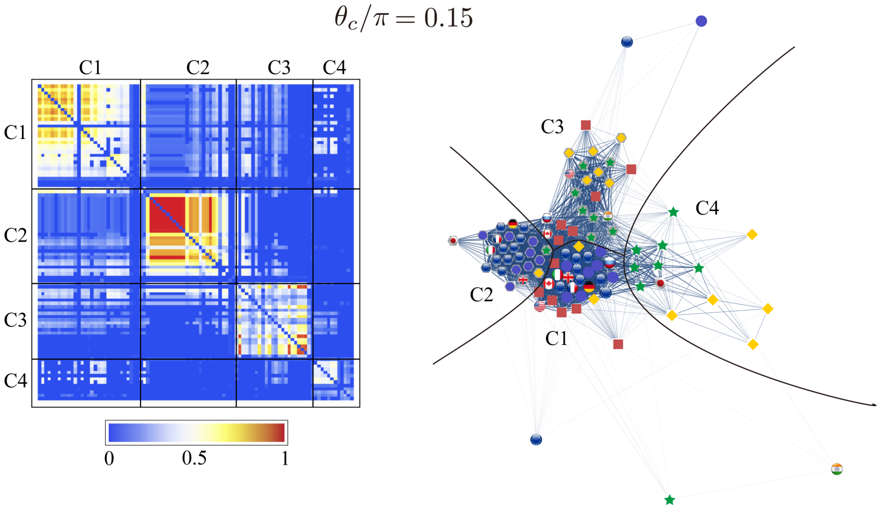

Figure D2. Community structure for $\theta_c = 0.15$. Raising θ_c by one and a half increases the number of links from 3,890 to 4,644, leading to a network in which nodes are more tightly connected. However, we still observe four communities organized in almost the same way as in Fig. 13.

References

1. Vodenska I, Aoyama H, Fujiwara Y, Iyetomi Y, Arai Y. Supplementary Information; 2014. http://www.econophysics.jp/download/vodenska_et_al_2014/index.html.
